# Confinement tonicity on epidemic spreading

**DOI:** 10.1007/s00285-024-02064-1

**Published:** 2024-03-22

**Authors:** Alexis Erich S. Almocera, Alejandro H. González, Esteban A. Hernandez-Vargas

**Affiliations:** 1 Department of Mathematics, Physics and Computer Science, College of Science and Mathematics, University of the Philippines Mindanao, Davao City, Philippines; 2 Institute of Technological Development for the Chemical Industry (INTEC), CONICET-Universidad Nacional del Litoral (UNL), Santa Fe, Argentina; 3 Department of Mathematics and Statistical Science, University of Idaho, Moscow, ID 83844-1103 USA; 4 Institute for Modeling Collaboration and Innovation, University of Idaho, Moscow, ID 83844-1103 USA

**Keywords:** Epidemic, Confinement, Stability, Epidemic final size, 92D30, 34D20, 34C60

## Abstract

Emerging and re-emerging pathogens are latent threats in our society with the risk of killing millions of people worldwide, without forgetting the severe economic and educational backlogs. From COVID-19, we learned that self isolation and quarantine restrictions (confinement) were the main way of protection till availability of vaccines. However, abrupt lifting of social confinement would result in new waves of new infection cases and high death tolls. Here, inspired by how an extracellular solution can make water move into or out of a cell through osmosis, we define confinement tonicity. This can serve as a standalone measurement for the net direction and magnitude of flows between the confined and deconfined susceptible compartments. Numerical results offer insights on the effects of easing quarantine restrictions.

## Introduction

Pandemics are latent threat to public health. In history, we have encountered the Black Death in the mid-1300s, the 1918 flu pandemic, H1N1 influenza pandemic in 2009, Ebola 2013 and 2018, Madagascar Plague outbreak 2014, Measleas outbreak 2019–2020, among many others (Hernandez-Vargas et al. 2019). In recent history, the 2019–2022 coronavirus pandemic (COVID-19) has made significant global impacts (Guo et al. 2020; Huang et al. 2020; Wang et al. 2020). Pandemics pose several challenges to determine effective disease control strategies tailored to a national or local region.

**Fig. 1:**
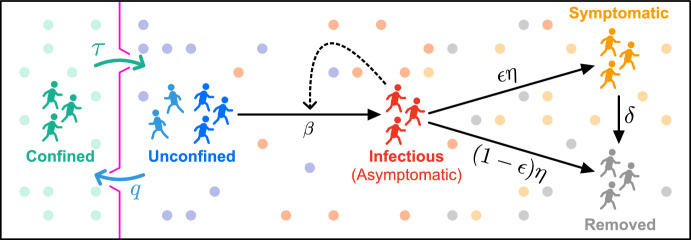
A schema representing our modeling framework, where freely-moving molecules (small circles) represent individuals. A semi-permeable membrane (pink) isolates the confined individuals (green) from the disease spread, inducing an “osmosis” given by the net confinement (blue arrow, with per-capita rate *q*) and deconfinement (green arrow, with per-capita rate $\tau$). The schema embeds a compartmental diagram describing the progression of unconfined individuals (blue) as they become infectious (red), symptomatic (orange), and finally removed from the population (gray)(color figure online)

A diverse collection of epidemic models was proposed to gain further quantitative understanding of the pandemic as it evolves and to emphasize the importance of disease control measures (Anderson et al. 2020; Ferguson et al. 2020; Heesterbeek et al. 2015; Mejia-Hernandez and Hernandez-Vargas 2020). A central measure to avoid infections involves quarantine (or confinement). SEIR minimal compartmental epidemic model to study the effects of sharp and gradual lifting of confinement has been formulated in Ricardo-Azanza and Hernandez-Vargas (2020), Peng et al. (2020) and López and Rodó (2020). Among all numerical results (Ricardo-Azanza and Hernandez-Vargas 2020; Peng et al. 2020), the models predict that implementing a sharp lifting of confinement strategies, i.e., immediate return to pre-COVID activities, would result in a massive peak in infected cases. This observation leads to the possibility of a breakdown in the health system due to a surge in patient admissions. Moreover, the model in Ricardo-Azanza and Hernandez-Vargas (2020) suggested a better strategy through phased gradual de-confinement tailored to each city in Mexico. Aside from these observations, no mathematical analysis was carried out on the SEIR model with confinement to determine qualitative features like stability and long-term behavior. We address this gap by associating the confinement behavior with osmosis between two substances (Fig. 1).

Model analysis based on dynamical systems theory can lead to a more in-depth understanding of the disease. For example, in-host COVID-19 modeling using dynamical systems and control theory helped to identify conditions for reducing the pathogen load (Almocera et al. 2020; Abuin et al. 2020). On a population-level, the severity of the disease (through the basic reproduction number), epidemic final sizes, and epidemic peak times have been established for previous epidemic models (Ricardo-Azanza and Hernandez-Vargas 2020; Arino et al. 2007). From a control-theoretic view, SIR modeling studies considered various approaches to reduce the epidemic peak size through maximal, long-term confinement (Bliman and Duprez 2021), single short-term and non-repeating (“one-shot”) actions (Di Lauro et al. 2021), and timed control of the contact rate over finite time (Ketcheson 2021).

We organize this paper as follows. After a brief review of epidemic terms in Sect. 2, the compartmental epidemic model SEIRC is presented in Sect. 3 to highlight the confinement and deconfinement of susceptible individuals. We analyze the model in Sect. 4 to characterize equilibrium points, compute the basic reproduction number, and obtain global asymptotic properties. Section 6 concludes the paper with an in-depth discussion on the mathematical results and possible directions for future work.

## Review of epidemic terms

We review some primary concepts in epidemiology (Keeling and Rohani 2008), namely the basic reproduction number, herd immunity, final size, and epidemic peak, to contextualize our analysis; see also Sereno et al. (2021), Sereno et al. (2021) and González et al. (2022). Hence, it is helpful to consider the basic SIR model of Kermack and McKendrick (1927), given in dimensionless form by the following equations:

1 $$\begin{aligned} \left\{ \begin{aligned} \frac{dS}{dt}&= -\mathcal {R}_0 SI, \\ \frac{dI}{dt}&= \mathcal {R}_0 SI - I, \\ \frac{dR}{dt}&= I. \end{aligned}\right. \end{aligned}$$

The state variables *S*, *I*, and *R* denote the fractions of the population that are susceptible to the disease, infected and capable of spreading the disease, and removed (recovered or dead) individuals, respectively.

We call $\mathcal {R}_0$ the **basic reproduction number** or the average number of new infections caused by a single infected host during the course of infection when introduced to a completely susceptible population. This quantity is one of the most fundamental concepts in epidemiology. Typical models like (1) predict disease spread within a population when $\mathcal {R}_0 > 1$, supporting the importance of $\mathcal {R}_0$ as a threshold quantity and metric for mitigation efforts (Anderson et al. 2020). Moreover, these models have $\mathcal {R}_0 = \beta /\eta$ where $\beta$ and $\eta$ denote the transmission and removal (recovery or death) rates with appropriate units. However, various approaches are available for the calculation of $\mathcal {R}_0$ (van den Driessche 2017).

**Herd immunity** is generally associated with the protection of a critical fraction of the population to impede disease spread. There are different notions of herd immunity based on the proportion of immune individuals (Fine 1993). Here, we consider herd immunity as a critical population size under which the number of infected individuals do not increase (Sereno et al. 2021). With the SIR model (1), we may consider the **herd immunity threshold** $S^*$ such that $I' < 0$ when $S < S^*$. In our work, we consider a slightly restricted version of herd immunity that only considers part of the susceptible individuals that are unconfined (*x*) with infectious individuals (*z*): we define this herd immunity threshold $\widetilde{x}$ in (14), which will satisfy the property that $z'/z \le 0$ (*z* decreases) when $x \le \widetilde{x}$.

There are two related ideas about “final size.” The first idea is **epidemic final size**, which informally denotes how many individuals experienced the infection during an outbreak (Bidari et al. 2016). For the SIR model (1) assuming $S(0) = 1$, the epidemic final size *Z* is given by the so-called **final-size relation** $Z = 1 - S_\infty = 1 - e^{-\mathcal {R}_0Z}$ where $S_\infty := \lim _{t\rightarrow \infty } S(t)$ (see Ma and Earn 2006; Sereno et al. 2021). The limit value $S_\infty$ expresses the asymptotic behavior of the number of susceptible cases, which leads to our second idea: we define the **final size** of a state variable *u* corresponding to an epidemic state $u_\infty := \lim _{t\rightarrow \infty } u(t)$. For example, $S_\infty$ is the final size of the susceptible population. This notation is used, for example, in Hsu and Roeger (2007).

Finally, given the number or size of infected individuals *I*(*t*), we define the **epidemic peak** (or the **infected peak prevalence**) as the maximum value of *I* for all $t \ge 0$. This value and the epidemic final size are two control objectives of interest from an epidemic perspective (Di Lauro et al. 2021).

## Epidemic mathematical model with confinement

The following model (Ricardo-Azanza and Hernandez-Vargas 2020) modifies the standard SEIR model with confinement effects:

2 $$\begin{aligned} \left\{ \begin{aligned} \frac{dS}{dt}&= -\frac{\beta A}{N}S - (qS - \tau C),\\ \frac{dC}{dt}&= qS - \tau C,\\ \frac{dA}{dt}&= \frac{\beta A}{N}S - \eta A,\\ \frac{dI}{dt}&= \epsilon \eta A - \delta I,\\ \frac{dR}{dt}&= (1-\epsilon )\eta A + \delta I. \end{aligned}\right. \end{aligned}$$

Here, the total population is $N = S + C + A + I + R$. This model assumes that infectious individuals are asymptomatic (*A*), since otherwise they are isolated, and either progress to become infected with symptoms (*I*) or are removed (*R*). Unconfined susceptible individuals (*S*) enter the confined sub-population (*C*) at a per-capita rate of *q* (1/week), while the deconfinement from *C* to *S* is represented by the per-capita rate $\tau$ (1/week). Standard epidemic parameters are the transmission rate $\beta$, which according to this model occurs between susceptible and asymptomatic, incubation rate $\eta$, and the removal rate of symptomatic individuals $\delta$; all these parameters are measured in units of 1/week. We assume that a fraction $\epsilon$ of asymptomatic individuals present symptoms (i.e., enter the *I* compartment), while the rest ($1-\epsilon$) bypass the symptomatic stage and are directly removed.

### Remark 3.1

The state variable *S* represents only a fraction of the *classical* susceptible population given in this context by $S + C$. In the same vein, classical infected population (which represents both infected and infectious) is given here by $A + I$ where only *A* is infectious.

We can nondimensionalize the system (2) to scale each compartment size. Since *N* is constant with $dN/dt = 0$, we scale our variables with

$$\begin{aligned} x(t)&:= \frac{S(t)}{N},&y(t)&:= \frac{C(t)}{N},&z(t)&:= \frac{A(t)}{N}, \\ u(t)&:= \frac{I(t)}{N},&v(t)&:= \frac{R(t)}{N},&t^*&:= \tau t. \end{aligned}$$

The dimensionless variables *x*, *y*, *z*, *u*, and *v* are the fractions of *N* that are unconfined (susceptible), confined, asymptomatic, symptomatic, and removed, respectively. Introduce the dimensionless parameter $\mu ^*:= \mu /\tau$ for each dimensional parameter $\mu$ from (2). Then dividing through each equation of the system (2) by $\tau N$ yields the following dimensionless form:

3 $$\begin{aligned} \left\{ \begin{aligned} x'&= -\eta ^*\mathcal {R}_0xz - (q^* x - y),\\ y'&= q^* x - y,\\ z'&= \eta ^*\mathcal {R}_0xz - \eta ^*z,\\ u'&= \epsilon \eta ^* z - \delta ^* u,\\ v'&= (1-\epsilon )\eta ^* z + \delta ^* u, \end{aligned}\right. \end{aligned}$$

where ${}^\prime = d/dt^*$. We also have

4 $$\begin{aligned} x + y + z + u + v&= 1. \end{aligned}$$

We call

5 $$\begin{aligned} \mathcal {R}_0 := \frac{\beta ^*}{\eta ^*} = \frac{\beta }{\eta } \end{aligned}$$

the **basic reproduction number** *in the absence of control interventions*.

### Remark 3.2

The dimensionless parameter $q^* = q/\tau$ expresses the strictness of the confinement. Indeed, $q^* = y/x = C/S$ at any equilibrium point (steady state), from which $q^* > 1$ (resp. $q^* < 1$) can indicate that there are more (resp. less) confined individuals than those unconfined. Hence, we may associate a strict confinement with $q^* > 1$ and a lenient confinement with $q^* < 1$.

Now, we are interested in the effects of confinement on the behavior of asymptomatic individuals *z*. We observe that the differential equations of *x*, *y*, *z* in the system (3) do not depend on *u* and *v*. Therefore, it is enough to study the system

6 $$\begin{aligned} \left\{ \begin{aligned} x'&= -\eta ^*\mathcal {R}_0xz - (q^* x - y),\\ y'&= q^* x - y,\\ z'&= \eta ^*(\mathcal {R}_0xz - z), \end{aligned}\right. \end{aligned}$$

which generates the solutions of (3) with

$$\begin{aligned} u(t^*)&= \exp (-\delta ^*t^*)\left[ u(0) + \epsilon \eta ^*\int _0^{t^*} z(\theta )\exp (\delta ^*\theta ) d\theta \right] , \\ v(t^*)&= 1 - \left[ x(t^*) + y(t^*) + z(t^*) + u(t^*)\right] . \end{aligned}$$

In this manner, we can visualize the behavior of (3) with the *three-dimensional* phase portrait of (6). Furthermore, Eq. (4) necessitates $x + y + z \le 1$. Thus, we choose our state space (or feasible region) for the system (6) to be

7 $$\begin{aligned} \mathcal {X}&:= \left\{ (x, y, z) \in \mathbb {R}^3_+ \mid x + y + z \le 1\right\} \end{aligned}$$

where $\mathbb {R}^3_+$ denotes the non-negative octant (i.e., the set of points in three-dimensional space with non-negative coordinates). Finally, we can quantify the net movement between the confined and unconfined compartments. We introduce a dimensionless quantity

8 $$\begin{aligned} Q(t^*)&:= q^* - \frac{y(t^*)}{x(t^*)} = \frac{y'(t^*)}{x(t^*)}. \end{aligned}$$

Based on an upcoming result on how *y* changes over time, we may call *Q* a **confinement tonicity** inspired by how water flows between two solutions undergoing osmosis. A central result of our work is a formula for the final size of the unconfined population that incorporates the integral of *Q* over all forward times.

## Mathematical analysis

### Equilibria

We begin our dynamical analysis by defining the equilibrium sets of the system (6). Any equilibrium point with coordinates (*x*, *y*, *z*) satisfies the following equations:

$$\begin{aligned} 0&= -\eta ^*\mathcal {R}_0xz - (q^* x - y),\\ 0&= q^* x - y,\\ 0&= \eta ^*\mathcal {R}_0xz - \eta ^* z. \end{aligned}$$

These equations yield $z = 0$ and $y = q^*x$. Restricted to the state space $\mathcal {X}$, we also have

$$\begin{aligned} x + y + z&= (1 + q^*)x \le 1&\implies{} & {} 0 \le x&\le \frac{1}{1+q^*}. \end{aligned}$$

Therefore, the set

9 $$\begin{aligned} \mathcal {X}^*&:= \left\{ (x, q^*x, 0)~\Bigg \vert ~0 \le x \le \frac{1}{1+q^*}\right\} , \end{aligned}$$

consists all the (non-isolated) equilibrium points of (6) in $\mathcal {X}$. Furthermore, the line segment connecting the origin and the point

10 $$\begin{aligned} E(q^*)&:= \left( \frac{1}{1+q^*}, \frac{q^*}{1+q^*}, 0\right) \end{aligned}$$

represents $$\mathcal {X}^*$$.

Observe that $E(q^*)$ corresponds to the steady state of (3) where $x+y = 1$ and $z = u = v = 0$. Hence, in the context of Remark 3.1, we may interpret $E(q^*)$ as the completely susceptible population. However, the disease-free scenario without the pathogen is only meaningful without confinement, that is, when viewing $q^*$ as control parameter and $q^* = 0$. Thus, we define the **disease-free equilibrium**

$$\begin{aligned} \text {DFE}&:= (1, 0, 0) = E(0) \end{aligned}$$

where $$y = 0$$.

#### Remark 4.1

Each point in $\mathcal {X}^*$ corresponds to the following equilibrium point of (3) with the same *x*-coordinate:

$$\begin{aligned} y&= q^*x,&z&= u = 0,&v = 1 - (1+q^*)x. \end{aligned}$$

Now, the equilibrium points in $\mathcal {X}^*$ are *non-isolated* in the sense that no open set separates any equilibrium point from the rest. Although we typically employ linearization for isolated equilibrium points, we can classify equilibrium points in $\mathcal {X}^*$ based on the Jacobian matrix. The Jacobian matrix of the system (6) is

$$\begin{aligned} J(x, y, z)&= \begin{bmatrix} - \eta ^*\mathcal {R}_0z - q^* &{} 1 &{} -\eta ^*\mathcal {R}_0x \\ q^* &{} -1 &{} 0 \\ \eta ^*\mathcal {R}_0z &{} 0 &{} \eta ^*(\mathcal {R}_0x - 1) \end{bmatrix}. \end{aligned}$$

Evaluating *J* at any point on $\mathcal {X}^*$, we obtain

$$\begin{aligned} J^*&= \begin{bmatrix} -q^* &{} 1 &{} -\eta ^*\mathcal {R}_0x \\ q^* &{} -1 &{} 0 \\ 0 &{} 0 &{} \eta ^*(\mathcal {R}_0x - 1) \end{bmatrix},&0&\le x \le \frac{1}{1+q^*}. \end{aligned}$$

The eigenvalues of $J^*$ are 0, $-(1+q^*)$, and

$$\begin{aligned} \lambda&= \eta ^*\mathcal {R}_0\left( x - \frac{1}{\mathcal {R}_0}\right) . \end{aligned}$$

Let

11 $$\begin{aligned} \widehat{x}&:= \min \left\{ \frac{1}{\mathcal {R}_0}, \frac{1}{1+q^*}\right\} . \end{aligned}$$

Observe that $\lambda < 0$ when $x < \widehat{x} \le 1/\mathcal {R}_0$, and $\lambda \le 0$ when $x = \widehat{x}$. We also have $\widehat{x} < x \le 1/(1+q^*)$ only if $\widehat{x} = 1/\mathcal {R}_0$, from which $\lambda > 0$. Thus we obtain the following partition of $\mathcal {X}^*$:

12 $$\begin{aligned} \mathcal {X}^*_\text {st}&:= \left\{ (x, q^*x, 0) \in \mathcal {X} \mid 0 \le x \le \widehat{x}\right\} , \end{aligned}$$

13 $$\begin{aligned} \mathcal {X}^*_\text {un}&:= \left\{ (x, q^*x, 0) \in \mathcal {X} \mid \widehat{x} < x \le 1/(1+q^*)\right\} . \end{aligned}$$

Based on the signs of the eigenvalues, we may call $\mathcal {X}^*_\text {st}$ and $\mathcal {X}^*_\text {un}$ the **stable and unstable sets**, respectively. Theorem 4.12(a) justifies this notation in the local stability of  $\mathcal {X}^*_\text {st}$.

Generally speaking, we define herd immunity as the threshold proportion of the unconfined population (*x*) below which the asymptomatic population (*z*) decreases. Since $\mathcal {R}_0$ does not change with the control parameter $q^*$, we consider the endemic case and define our **herd immunity threshold** as

14 $$\begin{aligned} \widetilde{x}&:= \frac{1}{\mathcal {R}_0} \end{aligned}$$

from which $\widehat{x} = \min \{\widetilde{x}, 1/(1+q^*)\}$. If $x \le \widetilde{x}$, then

15 $$\begin{aligned} \frac{z'}{z}&= \eta ^*\mathcal {R}_0\left( x - \frac{1}{\mathcal {R}_0}\right) \le \eta ^*\mathcal {R}_0\left( \widetilde{x} - \frac{1}{\mathcal {R}_0}\right) = 0, \end{aligned}$$

hence *z* decreases.

For a possible extension of our model that allows external action to change $\mathcal {R}_0$, we may redefine our herd immunity as $\widetilde{x} = \min \{1/\mathcal {R}_0, 1\}$. Then arguing as in (15), *z* decreases when $x \le \widetilde{x}$.

### Positively invariant sets

Next, we introduce results concerning invariant sets in $\mathcal {X}$ that are the natural generalization of equilibria. Henceforth, we call $D \subseteq \mathbb {R}^3$ a **positively invariant set** if any solution $\varphi$ of (6) with $\varphi (t_0) \in D$ for some $t_0 \ge 0$ satisfies $\varphi (t) \in D$ for all $t > t_0$. It is customary to call $\varphi$ a solution in *D*. The following theorem asserts that we may partition our state space $\mathcal {X}$ into three positively invariant sets.

#### Theorem 4.2

Write $$\mathcal {X} = \mathcal {Y}_1 \cup \mathcal {Y}_2 \cup \mathcal {Y}_3$$ where

$$\begin{aligned} \mathcal {Y}_1&= \{(x, y, z) \in \mathcal {X} \mid z = 0\}, \\ \mathcal {Y}_2&= \{(x, y, z) \in \mathcal {X} \mid z> 0\text { and }x = y = 0\}, \\ \mathcal {Y}_3&= \{(x, y, z) \in \mathcal {X} \mid z> 0,\text { and either }x> 0\text { or }y > 0\}. \end{aligned}$$

Then $\mathcal {Y}_k$ is positively invariant for $k = 1, 2, 3$, and $\mathcal {X}$ is positively invariant.

#### Proof

Note that positively invariant sets are closed under set union, hence, it suffices to establish the positive invariance of $\mathcal {Y}_k$ for $k = 1, 2, 3$. According to the system (6), the derivative $z' = 0$ whenever $z = 0$, which establishes the positive invariance of $\mathcal {Y}_1$. We have $x = y = 0$ in $\mathcal {Y}_2$, from which $x' = y' = 0$. Thus, $\mathcal {Y}_2$ is positively invariant.

Now, let $\pi _1$ and $\pi _2$, $\pi _3$ be the intersections of $\mathcal {Y}_3$ with the planes $x = 0$, $y = 0$, and $x + y + z = 1$, respectively. These planes have corresponding outward normal vectors $\vec {n}_1 = \langle {-1, 0, 0}\rangle$, $\vec {n}_2 = \langle {0, -1, 0}\rangle$, and $\vec {n}_3 = \langle {1, 1, 1}\rangle$. Moreover, we obtain the following negative dot products:

$$\begin{aligned} \left. \vec {n}_1 \cdot \langle {x', y', z'}\rangle \right| _{\pi _1}&= -y, \\ \left. \vec {n}_2 \cdot \langle {x', y', z'}\rangle \right| _{\pi _2}&= -q^* x, \\ \left. \vec {n}_3 \cdot \langle {x', y', z'}\rangle \right| _{\pi _3}&= -\eta ^*z. \end{aligned}$$

This result combined with the positive invariance of $\mathcal {Y}_1$ and $\mathcal {Y}_2$ implies that the direction vectors of (6) direct inwards on the boundary of $\mathcal {Y}_3$. Therefore, $\mathcal {Y}_3$ is positively invariant. $\square$

#### Remark 4.3

Solutions restricted to $\mathcal {Y}_1 = \mathcal {X} \cap \{(x, y, z) \in \mathbb {R}^3_+ \mid z = 0\}$ satisfy $x' + y' = 0$ and are thus implicitly defined by

$$\begin{aligned} x + y&= x(0) + y(0),&z&\equiv 0. \end{aligned}$$

Moreover, $x(t^*)$ and $y(t^*)$ *monotonically* approach their respective limits as $t^* \rightarrow \infty$. These limits are

$$\begin{aligned} \lim _{t^* \rightarrow \infty } x(t^*)&= \frac{x(0) + y(0)}{1 + q^*},&\lim _{t^* \rightarrow \infty } y(t^*)&= \frac{q^*[x(0) + y(0)]}{1 + q^*}. \end{aligned}$$

On $\mathcal {Y}_2$ where $x = 0$ and $y = 0$, the system (6) reduces to a single equation, $z' = -\eta ^*z$, from which $z(t^*) = z(0)\exp (-\eta ^*t^*) \rightarrow 0$ as $t^* \rightarrow \infty$.

#### Remark 4.4

Points on $\mathcal {Y}_3 \cap \{(x, y, z) \in \mathbb {R}^3_+ \mid x = 0\}$ satisfy $y > 0$, and thus, $x' = y > 0$. Similarly, solutions in $\mathcal {Y}_3$ with $y = 0$ satisfy $y' > 0$. Thus, solutions in the positive invariant set $\mathcal {Y}_3$ enter

$$\begin{aligned} \{(x, y, z) \in \mathcal {X} \mid x> 0, y> 0, z > 0\}, \end{aligned}$$

in forward time. That is, *solutions of* (6) *where* *z*(0) * and either* *x*(0) or *y*(0) * are positive eventually have positive coordinates*.

#### Remark 4.5

The positive invariance of $\mathcal {X}$ permits our choice of an initial point to be anywhere inside $\mathcal {X}$. In practice, however, we restrict our initial conditions to $\mathcal {Y}_3 \cap \{y = 0\}$ with

$$\begin{aligned} x(0)&= 1 - \varepsilon ,&y(0)&= 0,&z(0)&= \varepsilon ,&u(0)&= 0,&v(0)&= 0, \end{aligned}$$

where $0 < \varepsilon \ll 1$. These conditions are near the completely susceptible case where $x = 1$.

### Reproduction number with confinement strictness

We may compute another epidemic threshold based on the next-generation matrix method (van den Driessche 2017). Given that *z* is the only state variable in (6) that has infected individuals, we have

$$\begin{aligned} z'&= \mathcal {F}(x, y, z) - \mathcal {G}(x, y, z),&\mathcal {F}(x, y, z)&= \eta ^*\mathcal {R}_0xz,&\mathcal {G}(x, y, z)&= \eta ^*z. \end{aligned}$$

Here, $\mathcal {F}$ represents the inflow of new infections, and $\mathcal {G}$ represents the outflow of individuals from the *z* compartment. Let

$$\begin{aligned} F&= \frac{\partial \mathcal {F}}{\partial {z}} = \eta ^*\mathcal {R}_0\left( \frac{1}{1+q^*}\right) ,&G&= \frac{\partial \mathcal {G}}{\partial {z}} = \eta ^*, \end{aligned}$$

where the partial derivatives are evaluated at $E(q^*)$, where $z = 0$, and define $\mathcal {R}_C:= FG^{-1}$. Computing, we have

16 $$\begin{aligned} \mathcal {R}_C&= \frac{\mathcal {R}_0}{1 + q^*}. \end{aligned}$$

We may also characterize $\mathcal {R}_C$ with the following observation: $z'/z > 0$ at $E(q^*)$ if and only if $\mathcal {R}_C > 1$. For $q^* = 0$ (i.e., evaluating at DFE), $FG^{-1}$ becomes the ($1 \times 1$) next-generation matrix, and $\mathcal {R}_C = \mathcal {R}_0$. Moreover, $\mathcal {R}_C$ reduces with strict confinement (see Remark 3.2), hence we may call $\mathcal {R}_C$ a **reproduction number with confinement strictness**.

#### Remark 4.6

Equation (16) views $\mathcal {R}_C$ as a linear function of $\mathcal {R}_0$ and

$$\begin{aligned} \frac{d\mathcal {R}_C}{d\mathcal {R}_0}&= \frac{1}{1 + q^*} = \frac{\tau }{\tau + q}, \end{aligned}$$

hence every unit of change in $\mathcal {R}_0$ corresponds to a change of $1/(1+q^*)$ units in $\mathcal {R}_C$. Thus with a large $q^*$ (corresponding to either a large *q* or small $\tau$), marginal changes in $\mathcal {R}_C$ are small when compared to the corresponding changes $\mathcal {R}_0$.

### Asymptotic behavior

Here, we denote the following limits for simplicity:

$$\begin{aligned} x_{\infty }&:= \lim _{t\rightarrow \infty } x(t),&y_{\infty }&:= \lim _{t\rightarrow \infty } y(t),&z_{\infty }&:= \lim _{t\rightarrow \infty } z(t). \end{aligned}$$

We refer to these limits as final sizes, which may depend on the model parameters and the initial condition of the solution.

To establish the existence of these final sizes, we first note that the second derivative

17 $$\begin{aligned} y''&= q^*x' - y' = -q^*\eta ^*\mathcal {R}_0xz - (1 + q^*)y' \end{aligned}$$

determines the monotone properties of $y(t^*)$ in the following result.

#### Theorem 4.7

Consider a solution of (6) in the state space $\mathcal {Y}_3$ where $z(t_0) > 0$ for some $t_0 \ge 0$. Then the following statements are true:

- If $y'(t_1) = 0$ for some $t_1 \ge t_0$, then $y''(t_1) < 0$ (the only optimal value that *y* can reach is a maximum).
- If $y'(t_0) \le 0$, then $y'(t^*) < 0$ for all $t^* > t_0$ (if *y* does not increase for some time, then *y* decreases for all further time).
- If $y'(t_0) > 0$ and $y'(t_1) = 0$ for some $t_1 > t_0$, then we can choose $t_1$ such that $y'(t^*)$ has the same sign with $t_1 - t^*$ for all $t^* > t_0$ (if *y* increases and reaches an optimal value, then this value is the unique maximum value).

#### Proof

By virtue of Remark 4.4, we assume without loss of generality that $x(t^*) > 0$, $y(t^*) > 0$, and $z(t^*) > 0$ for all $t^* \ge t_0$. Then statement (a) is an immediate consequence of Eq. (17).

For statement (b), it is enough to consider $y'(t_0) < 0$, since $y'(t_0)=0$ implies $y''(t_0) < 0$ by statement (a), and $y'(t^*) < 0$ as $t^*$ increases from $t_0$. Given $y'(t_0) < 0$, suppose that there exists $t_1 > t_0$ such that $y'(t_1) \ge 0$. Then we choose $t_1$ to be the smallest such value that $y'(t_1) = 0$. However, statement (a) yields $y''(t_1) < 0$ (i.e., $y'$ decreases near $t_1$) and $y'(t^*) > 0$ as $t^* \rightarrow t_1$ from the left. We obtain a contradiction because our choice of $t_1$ forces $y'(t^*) < 0$ for $t_0 \le t^* < t_1$. Therefore, $y'(t^*) < 0$ for all $t > t_0$. We have proven statement (b).

We now prove statement (c) by supposing that $y'(t_0) > 0$ and $y'(t_1) = 0$ for some $t_1 > t_0$. Then we can take $t_1$ to be the smallest possible value such that $y'(t^*) > 0$ for all $t^* < t_1$. Then appealing to statement (b) where $t_1$ takes the place of $t_0$, we have $y'(t^*) < 0$ for all $t^* > t_1$. Therefore, $y'(t^*)$ takes equal sign with $t_1 - t^*$ for all $t^* > t_0$. $\square$

#### Corollary 4.8

For any solution $\varphi (t^*) = (x(t^*), y(t^*), z(t^*))$ of (6), the limit $y_{\infty }$ is a unique finite real number.

#### Proof

Consider the positively invariant sets $\mathcal {Y}_k$ ($k = 1, 2, 3$) in Theorem 4.2, which define a partition of our state space $\mathcal {X}$. Then by Remark 4.3, the limit $y_{\infty }$ uniquely exists whenever $\varphi$ is a solution in either $\mathcal {Y}_1$ or $\mathcal {Y}_2$. If $\varphi$ is a solution in $\mathcal {Y}_3$, then the hypotheses of Theorem 4.7 hold where $t_0 = 0$. We argue as follows:

- If $y'(0) \le 0$, then $y(t^*)$ strictly decreases for all $t^* > 0$ according to statement (b).
- If $y'(0) > 0$, then either $y(t^*)$ strictly increases for all $t^* > 0$ or it initially increases to a global maximum and finally decreases.

Therefore, $y(t^*)$ is monotone for sufficiently large $t^*$, which guarantees the existence of a unique $y_{\infty }$. Since the solution is restricted to the state space $\mathcal {X}$, which is a bounded set, $y_{\infty }$ is a unique finite real number. $\square$

#### Theorem 4.9

Consider a solution of (6) in $\mathcal {X}$ with initial value at $t^* = t_0 \ge 0$. Then $(x + y)(t^*)$ decreases, i.e., $(x+y)'(t^*) \le 0$, for all $t^* \ge t_0$. Moreover, $(x_{\infty }, y_{\infty }, z_{\infty }) \in \mathcal {X}^*_\text {st}$ when $z(t_0) > 0.$

#### Proof

Recalling the partition $\mathcal {X} = \mathcal {Y}_1 \cup \mathcal {Y}_2 \cup \mathcal {Y}_3$ in Theorem 4.2, our result holds for solutions in $\mathcal {Y}_1$ and $\mathcal {Y}_2$. Thus appealing to Remark 4.4, we assume without loss of generality that $x > 0$ and $z > 0$. Then the system (6) yields

$$\begin{aligned} (x+y)'(t^*) = -\eta ^*\mathcal {R}_0x(t^*)z(t^*) < 0 \end{aligned}$$

for all $t^* \ge t_0$. By Corollary 4.8, $x_{\infty } = (x+y)_{\infty } - y_{\infty }$ uniquely exists.

Now, the second derivative $y''$ in (17) expands into a polynomial expression in the solution coordinates, which inherit the finite bounds of the state space $\mathcal {X}$. Hence, $y'_{\infty } = q^*x_{\infty } - y_{\infty } = 0$ by Barbălat’s lemma, and $y_{\infty } = q^*x_{\infty }$. Similarly, Barbălat’s lemma also yields $(x+y)'_\infty = 0$, and

18 $$\begin{aligned} z'(t^*)&= - (x + y)'(t^*) - \eta ^*z(t^*) \approx -\eta ^*z(t^*). \end{aligned}$$

as $t^*\rightarrow \infty$. Moreover, $z(t^*) \approx C\exp (-\eta ^*t^*)$ for some $C > 0$ and sufficiently large values of $t^*$, hence $z_{\infty } = 0$. To complete the proof, we claim that $x_\infty \le \widehat{x}$ along the following arguments:

- The bounds of the state space $\mathcal {X}$ imply that $x_{\infty } + y_{\infty } + z_{\infty } \le 1$, which reduces to $(1 + q^*)x_{\infty } \le 1$ and $x_{\infty } \le 1/(1+q^*)$.
- Equation (18) holds for sufficiently large $t^*$, from which $z' = \eta ^*z(\mathcal {R}_0x - 1) \le 0$ and $x(t^*) \le 1/\mathcal {R}_0$. Taking $t^*\rightarrow \infty$, we obtain $x_\infty \le 1/\mathcal {R}_0$.

Therefore, $$x_\infty \le \min \{1/\mathcal {R}_0, 1/(1+q^*)\} = \widehat{x}$$ and $$(x_{\infty }, y_{\infty }, z_{\infty }) \in \mathcal {X}^*_\text {st}$$. $$\square $$

Theorem 4.9 asserts that each state with $z > 0$ will end up at some point in the stable set $\mathcal {X}^*_\text {st}$. However, the result does not necessarily determine whether $\mathcal {X}^*_\text {st}$ is asymptotically stable (see “Appendix A” for the definition).

An immediate consequence of Remark 4.3 (for $z = 0$) and Theorem 4.9 is given by the following result.

#### Corollary 4.10

Every solution of (6) in $\mathcal {X}$ with $x_{\infty } > 0$ satisfies

$$\begin{aligned} Q_{\infty }&:= \lim _{t^*\rightarrow \infty } Q(t^*) = q^* - \frac{y_{\infty }}{x_{\infty }} = 0. \end{aligned}$$

Thus, $$y_\infty = q^* x_\infty $$.

Consider the solution with initial values $x(t_0)$, $y(t_0)$ and $z(t_0)$, for some $t_0 \ge 0$. From (6), we have

$$\begin{aligned} \frac{x'}{x}&= -\eta ^*\mathcal {R}_0z - \left( q^* - \frac{y}{x}\right) = -\eta ^*\mathcal {R}_0z - Q, \end{aligned}$$

where *Q* is defined in Eq. (8). Integrating both sides, we obtain:

19 $$\begin{aligned} \ln \left[ \frac{x(t^*)}{x(t_0)}\right]&= - \eta ^*\mathcal {R}_0\int _{t_0}^{t^*} z(\theta )\,d\theta - \int _{t_0}^{t^*} Q(\theta )\,d\theta , \nonumber \\ x(t^*)&= x(t_0)e^{\left[ -\eta ^*\mathcal {R}_0\int _{t_0}^{t^*} z(\theta )\,d\theta - \int _{t_0}^{t^*} Q(\theta )\,d\theta \right] } \nonumber \\ x(t^*)&= x(t_0)\frac{e^{\left[ -\eta ^*\mathcal {R}_0\int _{t_0}^{t^*} z(\theta )\,d\theta \right] }}{e^{\left[ \int _{t_0}^{t^*} Q(\theta )\,d\theta \right] }} \end{aligned}$$

for $t^* \ge t_0$. Meanwhile, adding the equations in (6) yield

$$\begin{aligned} -\eta ^*z&= x' + y' + z', \end{aligned}$$

from which

20 $$\begin{aligned} - \eta ^*\int _{t_0}^{t^*} z(\theta )\,d\theta&= (x+y+z)(t^*) - (x+y+z)(t_0) \end{aligned}$$

Substituting (20) into (19), we have

21 $$\begin{aligned} x(t^*)&= \frac{x(t_0)e^{\left[ \mathcal {R}_0(x+y+z)(t^*) - \mathcal {R}_0(x+y+z)(t_0)\right] }}{e^{\left[ \int _{t_0}^{t^*} Q(\theta )\,d\theta \right] }} \end{aligned}$$

for $t^* \ge t_0$. Taking $t^* \rightarrow \infty$ in (21) and applying the identity

$$\begin{aligned} x_{\infty } + y_{\infty } + z_{\infty } = (1+q^*)x_{\infty } \end{aligned}$$

by Remark 4.3 or Theorem 4.9, we have

$$\begin{aligned} x_{\infty }&= x(t_0)\frac{e^{\left[ \mathcal {R}_0\,(1+q^*)x_{\infty } - \mathcal {R}_0(x+y+z)(t_0)\right] }}{e^{\int _{t_0}^{\infty } Q(\theta )\,d\theta }}, \end{aligned}$$

or

22 $$\begin{aligned} x_{\infty }e^{-\mathcal {R}_0\,(1+q^*)x_{\infty }}&= \frac{x(t_0)e^{-\mathcal {R}_0[x(t_0)+y(t_0)+z(t_0)]}}{e^{\int _{t_0}^{\infty } Q(\theta )\,d\theta }}. \end{aligned}$$

Equation (22) is a central result of our work, which defines the final size $x_{\infty }$ of the unconfined population under a constant level of confinement. That is, with $q^*$ constant, our model predicts that the unconfined population $x(t^*)$ will eventually reach the proportion $x_{\infty }$. We emphasize that Eq. (22) incorporates the integral of the confinement tonicity, i.e., the function *Q* defined in (8), evaluated over all future times from $t_0$. Hence, unlike conventional forms that only depend on initial conditions (Arino et al. 2007), an exact value of the epidemic final size may also depend on the solution trajectory.

#### Theorem 4.11

Consider a solution in $\mathcal {Y}_2$ or $\mathcal {Y}_3$ where $z > 0$, such that $x(t^*)$ strictly decreases for all $t^* \ge t_0$.

- If $x(t_0) < 1/\mathcal {R}_0$, then $z(t^*)$ strictly decreases for all $t^* \ge t_0$. Moreover, $z_{\infty }:= \lim _{t^*\rightarrow \infty } z(t^*) = 0$.
- If $x(t_0) > 1/\mathcal {R}_C$, then $z'(t_0) > 0$. Furthermore, if there exists $t_1 > t_0$ such that $x(t^*) > 1/\mathcal {R}_0$ for $t^* < t_1$ and $x(t^*) < 1/\mathcal {R}_0$ for $t^* > t_1$, then $z(t^*)$ initially and strictly increases to its global maximum $z(t_1)$ and finally decreases to zero.

#### Proof

From (6), we have

$$\begin{aligned} z'(t^*)&= \eta ^*[\mathcal {R}_0x (t^*) - 1]z(t^*),&t^*&\ge t_0. \end{aligned}$$

Let $\sigma (t^*) = \eta ^*[\mathcal {R}_0x(t^*) - 1]$, and note from our assumption that $x(t^*) < x(t_0)$ for all $t^* > t_0$.

If $x(t_0) < 1/\mathcal {R}_0$, then $\sigma (t_0) < 0$ and $z'(t^*) < \sigma (t_0)z(t^*)$ for all $t^* > t_0$. Hence, $z(t^*)$ is strictly decreasing and $z_{\infty } = 0$. We have established statement (a).

If $x(t_0) > 1/\mathcal {R}_C$, then $\sigma (t_0)> \eta ^*(\mathcal {R}_Cx(t_0) - 1) > 0$ because $\mathcal {R}_C < \mathcal {R}_0$. Hence, we have $z'(t_0) = \sigma (t_0)z(t_0) > 0$. Now, suppose that there exists $t_1 > t_0$ such that $x(t^*) > 1/\mathcal {R}_0$ for $t^* < t_1$ and $x(t^*) < 1/\mathcal {R}_0$ for $t^* > t_1$. Then $\sigma (t^*) > 0$ and $z'(t^*) = \sigma (t^*)z(t^*) > 0$ for $t_0 \le t^* < t_1$. The global maximum occurs at $t^* = t_1$ where $z' = \sigma (t_1)z(t^*) = 0$. Applying statement (a) with any $t^* > t_1$ in place of $t_0$, we conclude that $z(t^*)$ initially and strictly increases to its global maximum $z(t_1)$ and finally decreases to zero. We have proven statement (b). $\square$

#### Theorem 4.12

For some $t_0 \ge 0$, fix the model parameter values, and consider

23 $$\begin{aligned} \zeta (x(t_0), y(t_0), z(t_0))&:= \frac{-\mathcal {R}_0\,(1+q^*)x(t_0)e^{-\mathcal {R}_0[x(t_0)+y(t_0)+z(t_0)]}}{e^{\int _{t_0}^{\infty } Q(\theta )\,d\theta }} \end{aligned}$$

Then the following statements hold:

- The stable set $\mathcal {X}^*_\text {st}$ is locally stable.
- If $-e^{-1} \le \zeta \le 0$ and $\mathcal {R}_0(1+q^*)x_\infty < 1$ over all solutions in the state space $\mathcal {X}$, then
  24 $$\begin{aligned} \mathcal {X}^*_{\textit{as}}&:= \left\{ (x,q^*x,0)~\Bigg \vert ~0 \le x \le \frac{\widetilde{x}}{1+q^*}\right\} \end{aligned}$$
  is an asymptotically stable subset of $\mathcal {X}^*_\text {st}$.
- Assuming that $\mathcal {X}^*_{\textit{as}}$ is asymptotically stable, suppose that, for every $p_0 = (x_0, q^*x_0, 0) \in \mathcal {X}^*_{\textit{as}}$ and arbitrary $\varepsilon$ with $0 < \varepsilon \ll 1$, there exist initial states $p_1$ and $p_2$ not in $\mathcal {X}^*$ such that $\Vert p_k - p_0\Vert < \varepsilon$ for each *k* and $\zeta (p_1) \ne \zeta (p_2)$, then $\mathcal {X}^*_{\textit{as}}$ is the smallest asymptotically stable set.

See “Appendix A” for the definitions of local and asymptotic stability.

#### Proof

To prove statement (a), consider any equilibrium point $x^* = (\xi , q^*\xi , 0) \in \mathcal {X}^*_\text {st}$ where $0 < \xi \le \widehat{x}$. Define

25 $$\begin{aligned} V(x, y, z)&= \left[ x - \xi - \xi \ln \left( \frac{x}{\xi }\right) \right] + \left[ y - q^*\xi - q^*\xi \ln \left( \frac{y}{q^*\xi }\right) \right] + z \end{aligned}$$

on the region where $x > 0$, $y > 0$, and $z \ge 0$, denoted $\mathcal {D}$. Then $V > 0$ on $\mathcal {D}-\{x^*\}$, and $V = 0$ at $x^*$. We compute $dV/dt^*$ below:

$$\begin{aligned} \frac{dV}{dt^*}&= \frac{\partial {V}}{\partial {x}}\cdot \frac{dx}{dt^*} + \frac{\partial {V}}{\partial {y}}\cdot \frac{dy}{dt^*} + \frac{\partial {V}}{\partial {z}}\cdot \frac{dz}{dt^*} \\&= \left( \frac{\xi }{x} - 1\right) (\eta ^*\mathcal {R}_0xz + q^* x - y) + \left( 1 - \frac{q^*\xi }{y}\right) (q^* x - y) + \eta ^*(\mathcal {R}_0x - 1)z \\&= \left[ \left( \frac{\xi }{x} - 1\right) \mathcal {R}_0x + (\mathcal {R}_0x - 1)\right] \eta ^*z + \left[ \left( \frac{\xi }{x} - 1\right) + \left( 1 - \frac{q^*\xi }{y}\right) \right] (q^* x - y) \\&= (\xi \mathcal {R}_0 - 1)\eta ^*z + \xi \left( \frac{1}{x} - \frac{q^*}{y}\right) (q^* x - y) \\&= (\xi \mathcal {R}_0 - 1)\eta ^*z - \frac{\xi (y - q^*x)^2}{xy} \end{aligned}$$

Since $\xi \le \widehat{x}$, we have $\xi \mathcal {R}_0 \le 1$ and thus $dV/dt^* \le 0$ on $\mathcal {D}$. Therefore, *V* is a Lyapunov function and $x^*$ is locally stable (see Theorem A.5). With $x^*$ arbitrary in $\mathcal {X}^*_\text {st}$, it follows that $\mathcal {X}^*_\text {st}$ and its subset $\mathcal {X}^*_{\textit{as}}$ are locally stable sets. This proves statement (a).

For statement (b), assume that $\zeta$ attains a maximum $\zeta = 0$ and a minimum of $\zeta = -e^{-1}$ over all solutions in the state space $\mathcal {X}$. Now, express (22) as the transcendental equation $\zeta = we^w$ where $w = -\mathcal {R}_0\,(1 + q^*)x_{\infty }$. Then $-1< w < 0$ and we have

26 $$\begin{aligned} w&= \mathcal {W}(\zeta )&\implies{} & {} x_\infty&= -\frac{\mathcal {W}(\zeta )}{\mathcal {R}_0(1+q^*)} \end{aligned}$$

where $\mathcal {W}$ is the principal branch of the Lambert function on a restricted domain $-e^{-1} \le \zeta \le 0$, where $\mathcal {W}$ strictly increases. Moreover, we have the following equivalent inequalities independent of any solution:

$$\begin{aligned} -1&\le \mathcal {W}(\zeta ) \le 0,&0&\le x_\infty \le \frac{1}{\mathcal {R}_0(1+q^*)}. \end{aligned}$$

Remark 4.3 and Theorem 4.9 additionally provide $0 \le x_\infty \le 1/(1+q^*)$, from which $0 \le x_\infty \le \min \{1/\mathcal {R}_0, 1\}/(1+q^*) = \widetilde{x}/(1+q^*)$. Therefore, $\mathcal {X}^*_{\textit{as}}$ is attractive.

Note that $\widetilde{x}/(1+q^*) \le \widehat{x}$, hence $\mathcal {X}^*_{\textit{as}} \subseteq \mathcal {X}^*_\text {st}$ (with equality if and only if $0 < \mathcal {R}_0 \le 1$). Therefore, $\mathcal {X}^*_{\textit{as}}$ inherits the local stability of $\mathcal {X}^*_\text {st}$ and becomes asymptotically stable. We have proven statement (b).

For statement (c), we consider the initial points $p_1$ and $p_2$ per assumption. For each, let the solution with initial point $p_k$ have the final size

$$\begin{aligned} x_{\infty , k}&= -\frac{\mathcal {W}(\zeta (p_k))}{\mathcal {R}_0(1+q^*)}. \end{aligned}$$

Then $\mathcal {W}(\zeta (p_1)) \ne \mathcal {W}(\zeta (p_2))$ and $x_{\infty , 1} \ne x_{\infty , 2}$ because $\mathcal {W}$ is injective. Thus, the solutions corresponding to the initial points $p_1$ and $p_2$ must converge to different points in $\mathcal {X}^*_{\textit{as}}$. Hence, *singleton and proper subsets* of $\mathcal {X}^*_{\textit{as}}$ are locally stable but not attractive. Therefore, $\mathcal {X}^*_{\textit{as}}$ is the smallest asymptotically stable set. $\square$

#### Remark 4.13

**Fig. 2:**
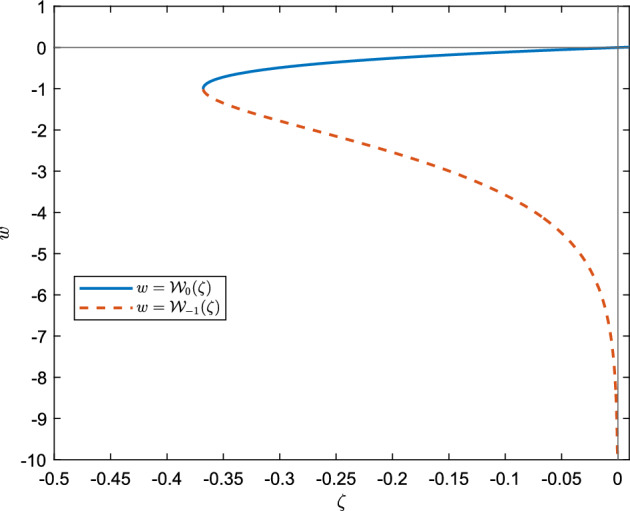
Two branches of the Lambert function, $w = \mathcal {W}_k(\zeta )$. Blue, solid line: $k = 0$ (principal branch). Red, dashed line: $k = -1$. The graphs of these functions meet at $\zeta = -e^{-1}$ where $w = -1$ (color figure online)

The final size $x_{\infty }$ depends on the parameters $\mathcal {R}_0$ and $q^*$. Thus, we can think of $w = -\mathcal {R}_0(1+q^*)x_\infty > -1$ as a constraint of the parameter space over all solutions. Since $(x_\infty , y_\infty , z_\infty ) \in \mathcal {X}_\text {st}^*$ and thus $(1+q^*)x_\infty \le 1$, it suffices (but is not necessary) that $\mathcal {R}_0 < 1$ for the constraint to apply. Removing the constraint may yield $w < -1$ for some solutions and $w > -1$ for others (see Fig. 13). Hence, we generally have two versions for the final size of a given solution:

$$\begin{aligned} x_\infty&= -\frac{\mathcal {W}_0(\zeta )}{\mathcal {R}_0(1+q^*)},&x_\infty&= -\frac{\mathcal {W}_{-1}(\zeta )}{\mathcal {R}_0(1+q^*)}, \end{aligned}$$

where $\mathcal {W}_0$ (principal branch) and $\mathcal {W}_{-1}$ are two branches of the Lambert function; see Fig. 2. If $w \ge -1$, then we take $\mathcal {W} = \mathcal {W}_0$; otherwise, we take $\mathcal {W} = \mathcal {W}_{-1}$.

#### Remark 4.14

The Lyapunov function *V* defined in Eq. (25) does not ensure asymptotic stability of single equilibrium points $x^* \in \mathcal {X}^*_\text {st}$ because $dV/dt = 0$ for all points where $z = 0$ and $y = q^*x$.

### Final size bounds

Consider the solution of our model, Eq. (6), with the following conditions where $t_0$ denotes initial time:

- $z(t_0) > 0$ and $x(t_0) > 0$, that is, both unconfined and asymptomatic populations have positive sizes.
- $y'(t_0) < 0$ or there exists $t_1 \ge t_0$ such that $y'(t_1) = 0$. That is, the confined population decreases or, per Theorem 4.7, reaches its maximum value at $t_1$.
- The improper integral $\int _{t_0}^{\infty } Q(\theta )\,d\theta$ is a finite real number.

Then

$$\begin{aligned} \zeta&< \widetilde{\zeta } \le \widehat{\zeta } \end{aligned}$$

where

27 $$\begin{aligned} \widetilde{\zeta }&:= -\frac{\mathcal {R}_0\,(1+q^*)x(t_0)e^{-\mathcal {R}_0[x(t_0)+y(t_0)+z(t_0)]}}{e^{\int _{t_0}^{t_1} Q(\theta )\,d\theta }}, \nonumber \\ \widehat{\zeta }&:= -\frac{\mathcal {R}_0\,(1+q^*)x(t_0)e^{-\mathcal {R}_0[x(t_0)+y(t_0)+z(t_0)]}}{e^{Q(t_0)(t_1-t_0)}}. \end{aligned}$$

Moreover, the final size relation given by

$$\begin{aligned} x_\infty&= -\frac{\mathcal {W}_k(\zeta )}{\mathcal {R}_0(1+q^*)},&\zeta&> -e^{-1},&k&\in \{0, -1\}, \end{aligned}$$

yields the following bounds:

- $$x_\infty > \widetilde{x}_\infty \ge \widehat{x}_\infty $$ for $$k = 0$$, and
- $$x_\infty < \widetilde{x}_\infty \le \widehat{x}_\infty $$ for $$k = -1$$,

where

28 $$\begin{aligned} \widetilde{x}_\infty&:= -\frac{\mathcal {W}_k(\widetilde{\zeta })}{\mathcal {R}_0(1+q^*)},&\widehat{x}_\infty&:= -\frac{\mathcal {W}_k(\widehat{\zeta })}{\mathcal {R}_0(1+q^*)}. \end{aligned}$$

See “Appendix B” for the derivations.

## Numerical results

The original parameters are given (Ricardo-Azanza and Hernandez-Vargas 2020) by

$$\begin{aligned} q&= 1,&\tau&= \frac{3}{5},&\eta&= \frac{1}{0.55},&\delta&= 2,&\epsilon&= 0.2, \end{aligned}$$

from which we compute the following **default parameter values** for our dimensionless models, systems (3) and (6):

$$\begin{aligned} \eta ^*&= \frac{1}{0.33} \approx 3.0303,&q^*&= \frac{5}{3} \approx 1.6667,&\delta ^*&= \frac{10}{3} \approx 3.3333. \end{aligned}$$

Note that we have a one-to-one correspondence between $\mathcal {R}_0$ and $\beta$, which permits a variation of $\mathcal {R}_0$ while leaving other dimensionless parameters fixed.

### Phase portraits

We adopt the following visual language to all our phase portraits:

- Solution trajectories appear as blue lines. The endpoints of each trajectory are empty and filled blue circles, corresponding to initial and final times respectively. The plot assumes $t^* = t_0 = 0$ for the initial time and $t^* = 10^4$ for the final time.
- The stable set $\mathcal {X}^*_\text {st}$ and the unstable set $\mathcal {X}^*_\text {un}$ appear as thick lines colored red and green, respectively.
- There are three semi-transparent triangular planes: $x+y+z=1$ (gray), $x=\widehat{x}$ (yellow), and $x=1/\mathcal {R}_0$ (blue). In the case $\widehat{x} =1/\mathcal {R}_0$, the blue triangle represents $x=\widehat{x} =1/\mathcal {R}_0$ (coinciding with the yellow triangle).

**Fig. 3:**
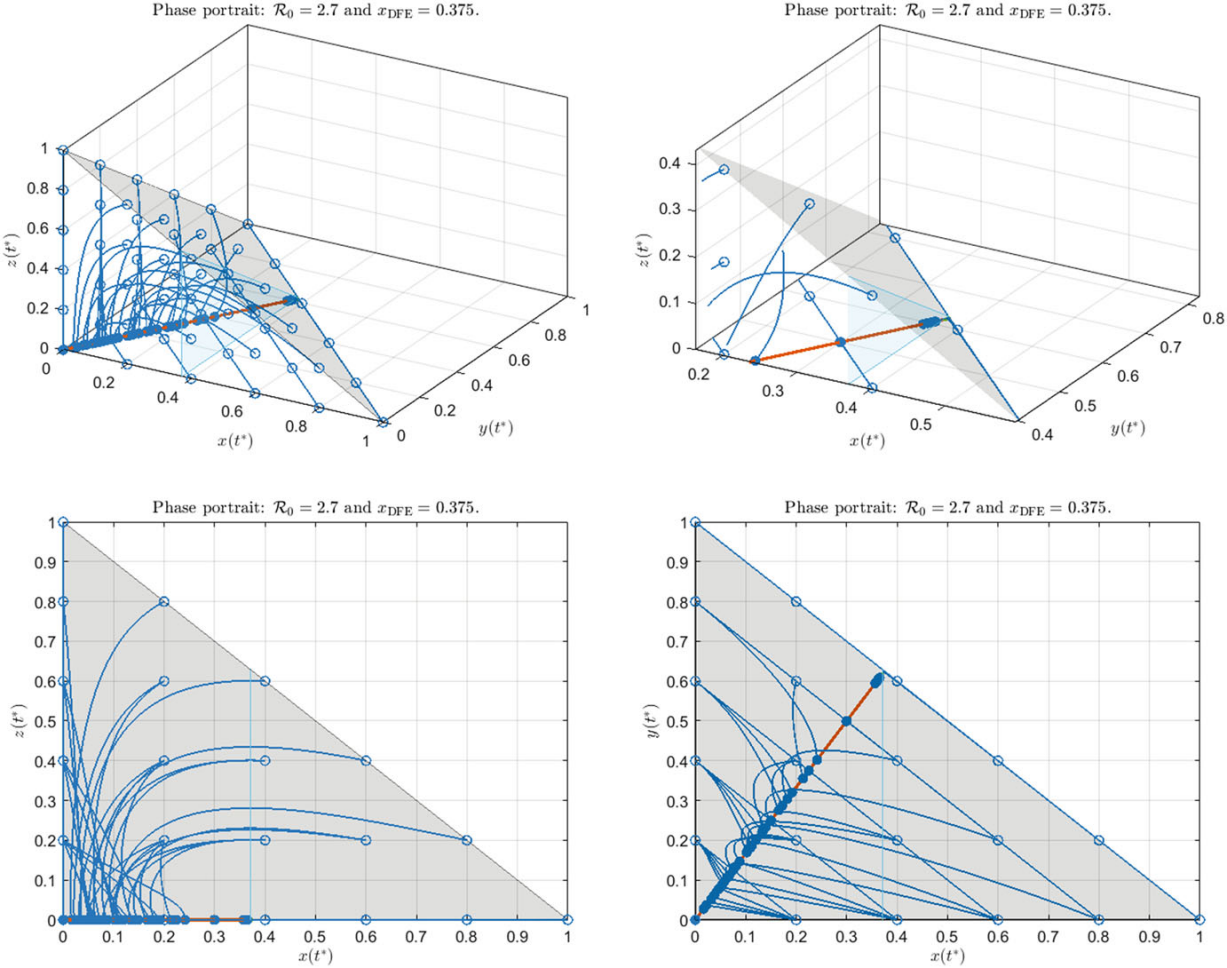
Phase portrait at different viewing angles with $\mathcal {R}_0 = 2.7$, $q^* = 5/3$, and $\eta ^* = 1/0.33$. Top left: three-dimensional view. Top right: magnification to the unstable set. Bottom left: projection to the *xz*-plane. Bottom right: projection to the *xy*-plane. Note that $z' = 0$ on $x = \widehat{x} = 1/\mathcal {R}_0$ (light blue triangular plane) and $y' = 0$ along $y = q^*x$. Not shown: the x-nullcline $\left( \eta ^* \mathcal {R}_0 z+q^* \right) x-y=0$ where $x^{\prime } =0$ (color figure online)

Figure 3 depicts the phase portrait generated with these default parameter values. We observe the following sequence of behaviors for a solution with initial point $x(t_0) > \widehat{x}$:

1. $x(t^*)$ strictly decreases, and both $y(t^*)$ and $z(t^*)$ strictly increases until some $t^* = t_1^*$ where $x(t_1^*) = \widehat{x}$ and $z'(t_1^*)=0$. From here, *z* decreases for $t^* > t_1^*$.
2. Either $y(t_1^*)$ will strictly increase for $t^* > t_1^*$ or the strict increase still occurs until some $t^* = t_2^*$ where $y(t_2^*) = q^* x(t_2^*)$ and $y'(t_2^*) = 0$.
3. Finally, if $y'(t_2^*) = 0$, then $y(t^*)$ strictly decreases for $t^* > t_2^*$.

Ultimately, each solution converges to some point $(x_\infty , y_\infty , 0) \in \mathcal {X}^*_\text {st}$. Furthermore, it is possible for $x'(t_3^*) = 0$ for some $t_3^* > t_2^*$ and $x(t_3^*)$ strictly *increases* for $t^* > t_3^*$ provided the solution trajectory intersects the nonplanar surface $y=\left( \eta ^* \mathcal {R}_0 z+q^* \right) x$ at $t^* =t_3^*$. We might attribute this effect to deconfinement.

**Fig. 4:**
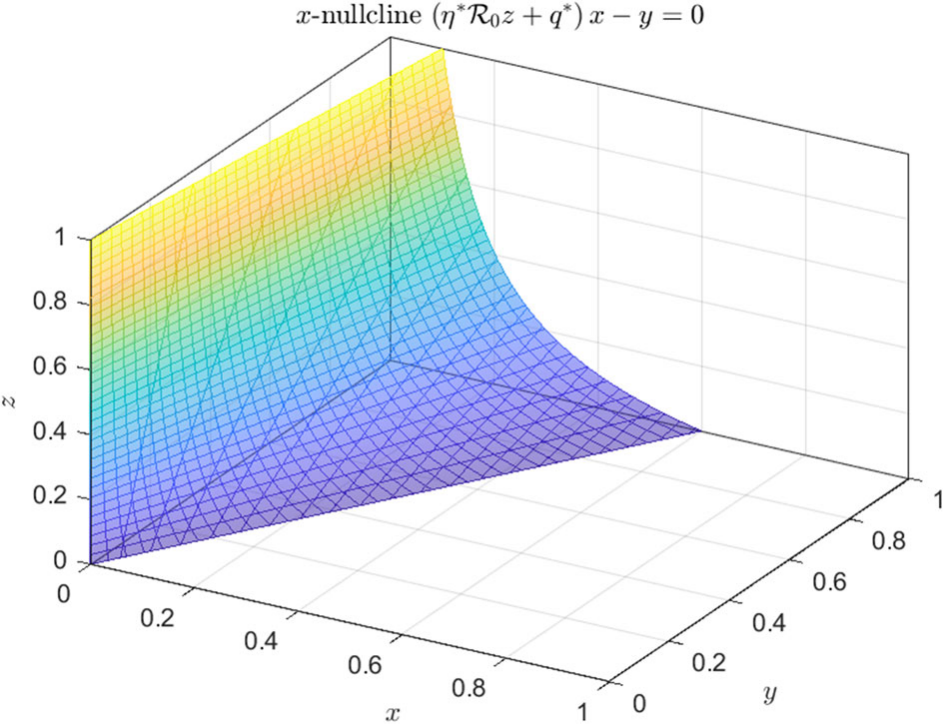
The *x*-nullcline surface where $x' = 0$. The equilibrium set $\mathcal {X}^*$ lies on the intersection of this surface with the *xy*-plane. Parameter values used: $\mathcal {R}_0 = 2.7$, $q^* = 5/3$, and $\eta ^* = 1/0.33$

These numerical observations warrant further analysis on the nullcline surfaces, direction vectors along these surfaces, and locating the final-size points. In particular, we identify the *x*-nullcline with the surface $(\eta ^* \mathcal {R}_0 z+q^*)x - y = 0$ on which $x' = 0$; see Fig. 4. Intricate qualitative dynamics might be associated with the nonplanar shape of this nullcline.

**Fig. 5:**
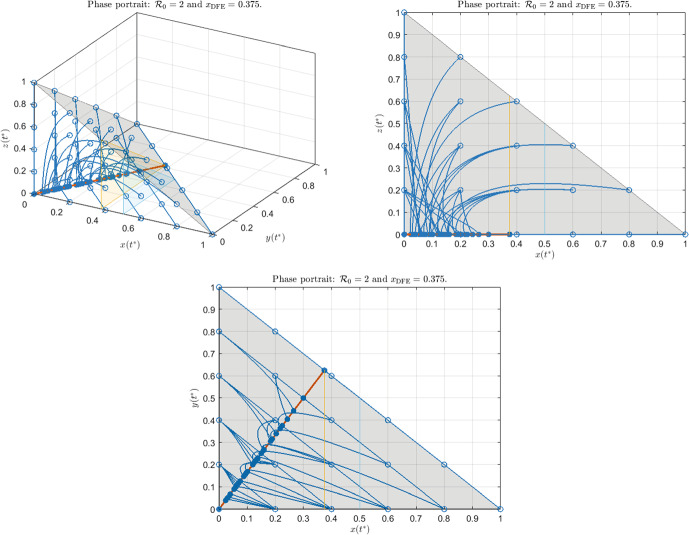
Phase portrait at different viewing angles with $\mathcal {R}_0 = 2$, $q^* = 5/3$, and $\eta ^* = 1/0.33$. Top-left: three-dimensional view. Top-right: projection onto the *xz*-plane. Bottom: projections onto the *xy*-plane. Both the planes $x = 1/\mathcal {R}_0$ (blue) and $x = \widehat{x} = 1/(1+q^*)$ (yellow) appear on the phase portrait. Only the stable set (thick red line) appears (color figure online)

**Fig. 6:**
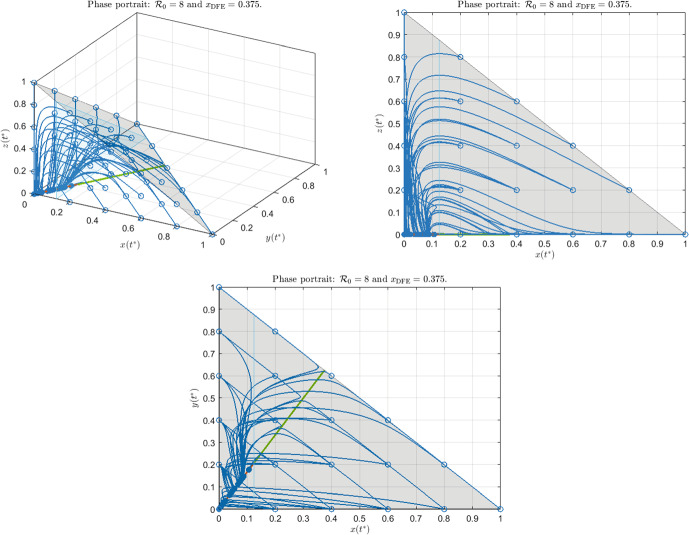
Phase portrait at different viewing angles with $\mathcal {R}_0 = 8$, $q^* = 5/3$, and $\eta ^* = 1/0.33$. Top-left: three-dimensional view. Top-right: projection onto the *xz*-plane. Bottom: projections onto the *xy*-plane. Note that the blue triangle represents both $x = 1/\mathcal {R}_0$ and $x=\widehat{x}$ because $1/\mathcal {R}_0 = 0.125 < 1/(1+q^*)$ (color figure online)

We have two cases when $\mathcal {R}_0 > 1$ according to $\widehat{x}$:

- Case 1, $\widehat{x} = 1/(1+q^*) >1/\mathcal {R}_0$: Fig. 5 illustrates this case for $\mathcal {R}_0 = 2$ with noticeable peaks in *y*. For solutions with initial point at $x<1/\mathcal {R}_0$, $z(t^*)$ initially increases to its global maximum value; cf. Fig. 3 ($\mathcal {R}_0 = 2.7$).
- Case 2, $\widehat{x} =1/\mathcal {R}_0 > 1/(1+q^*)$: Fig. 6 illustrates this case for $\mathcal {R}_0 = 8$. Here, both the stable and unstable sets are nonempty, and the stable set decreases in size when $\mathcal {R}_0$ increases further. We also observe that some solutions with $y(t_0) > q^*x(t_0)$ have $x(t^*)$ attain local maximum, possibly followed by a local minimum. Solutions with $y(t_0) < q^* x(t_0)$ cross $y = q^*x$ and remain in the region $y > q^*x$; these solutions experience a peak in *y*. For all solutions, $z(t^*)$ eventually decreases after possibly attaining a global maximum.

**Fig. 7:**
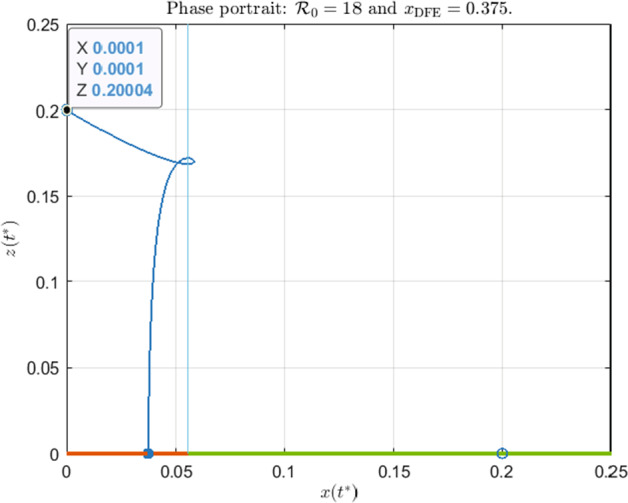
A solution trajectory (blue line) where $x(t_0) = y(t_0) = 0.0001$ and $z(t_0) = 0.20004$ for some $t_0 > 0$ (empty circle with data tip). Parameter values: $\mathcal {R}_0 = 18$, $q^* = 5/3$, $\eta ^* = 1/0.33$. The solution crosses $x = 1/\mathcal {R}_0$ (light-blue vertical line) twice, forming a small loop, before it ultimately tends to the stable set (thick red line) (color figure online)

We may theoretically consider larger values of $\mathcal {R}_0$ to anticipate possible variants that are more transmissible. Figure 7 suggests that larger values of $\mathcal {R}_0$ may introduce solutions with exceptional behavior. Here, $z(t^*)$ attains a local minimum, followed by a local maximum, before eventually decreasing to zero. This numerical example also indicates the non-monotone behavior of $x(t^*)$ due to the presence of the expression $q^*x - y$ in the corresponding differential equation.

### Stability and final size

The key results from Theorem 4.12 are the local stability of set $\mathcal {X}^*_\text {st}$ and the asymptotic stability of its subset $\mathcal {X}^*_{\textit{as}}$. These results hinge on the quantity $\zeta$ in Eq. (23) as a function of the initial point $(x(t_0), y(t_0), z(t_0))$. Equation (26) further establishes that $x_\infty$ is both a function of the same initial point and a decreasing function of $\zeta$. Since the principal Lambert function $\mathcal {W}$ increases over its restricted domain $-e^{-1} \le \zeta \le 0$, the final size $x_\infty$ attains its global maximum if and only if $\zeta$ attains its global minimum.

**Fig. 8:**
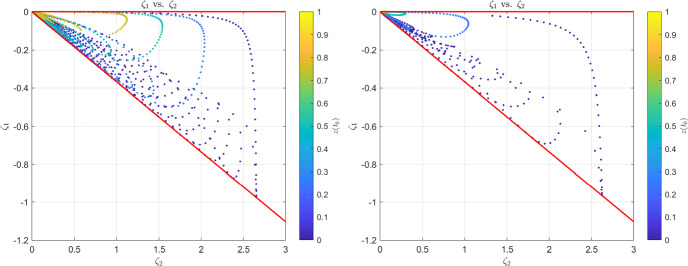
Numerical confirmation of the uniform bound of $\zeta = \zeta _1/\zeta _2$ over all solutions in $\mathcal {X}$ with $z > 0$, where $\zeta _1$ and $\zeta _2$ are defined in Eqs. (29)–(30). Left panel: $\mathcal {R}_0 = 2.7$; Right panel: $\mathcal {R}_0 = 8$. Both panels assume $\eta ^* = 3.0303$, $q^* = 1.6667$, and $t_0 = 0$. The points are generated from a lattice of initial points $(x(t_0), y(t_0), z(t_0))$ and colored according to the value of $z(t_0)$. These points are bounded by $\zeta _1 = 0$ and $\zeta _1 = -e^{-1}\zeta _2$ (thick solid red lines), implying the uniform bound $-e^{-1} \le \zeta \le 0$ (color figure online)

Now, we numerically verify Theorem 4.12(b) in Fig. 8. Here, we express $\zeta = \zeta _1/\zeta _2$ where

29 $$\begin{aligned} \zeta _1&:= -\mathcal {R}_0\,(1+q^*)x(t_0)e^{-\mathcal {R}_0[x(t_0)+y(t_0)+z(t_0)]}, \end{aligned}$$

30 $$\begin{aligned} \zeta _2&:= e^{\int _{t_0}^{\infty } Q(\theta )\,d\theta }. \end{aligned}$$

Each point in the figure represents a solution whose initial point is chosen from a lattice of sample points. The location $(\zeta _2, \zeta _1)$ of this point is above the line $\zeta = -e^{-1}$ and below the line $\zeta = 0$, implying that $-e^{-1} \le \zeta \le 0$.

Note that $\zeta _1$ corresponds to the epidemic final size at an equilibrium point, or equivalently, when the confinement tonicity is identically zero. Indeed, $y = q^*x$ and $Q = 0$ at any equilibrium point, from which $\zeta _2 = 1$ and $\zeta _1 = \zeta$. Moreover, $\zeta _1$ agrees with the classic final size relation which only needs initial population sizes to compute the exact value. In contrast, knowledge of the solution in forward time—and not only initial time—is necessary to calculate $\zeta _2$. Finding an exact non-integral form of $\zeta _2$ remains an open problem, but we present some attempts along this direction in Sect. 6.

**Fig. 9:**
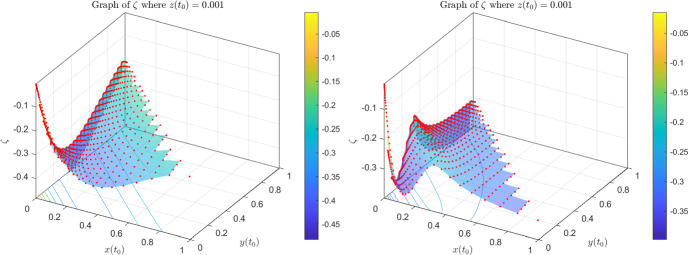
Graph of the function $\zeta$ generated by surface interpolation of $(x(t_0), y(t_0))$ (red points) with a fixed value $z(t_0) = 0.001$. Left panel: $\mathcal {R}_0 = 2.7$; Right panel: $\mathcal {R}_0 = 8$. Both panels assume $\eta ^* = 3.0303$, $q^* = 1.6667$, and $t_0 = 0$ (color figure online)

Figure 9 depicts the graph of $\zeta (x(t_0),y(t_0))$ where $z(t_0)$ is fixed. Here, it is possible to attain the global minimum $\zeta _{\min }$ of $\zeta$ not at a point but *along a curve* of initial points $(x(t_0), y(t_0))$. Furthermore, large values of $\mathcal {R}_0$ may introduce local maximum points along another curve. It is also possible to attain the global maximum at $\zeta = 0$ when $(x(t_0), y(t_0))$ approaches the origin.

The same figure suggests computing $\zeta _\text {min}$ as follows: for each $a \in [0, 1]$, let

$$\begin{aligned} \zeta _a&:= \min \{\zeta (x(t_0), y(t_0), z(t_0)) \mid z(t_0) = a\text { and }(x(t_0), y(t_0), z(t_0)) \in \mathcal {X}\}. \end{aligned}$$

Then $\zeta _{\min } = \min \{\zeta _a \mid 0 \le a \le 1\}$ and $x_\infty$ attains the maximum value $-\mathcal {W}(\zeta _{\min })/[\mathcal {R}_0(1+q^*)]$.

**Fig. 10:**
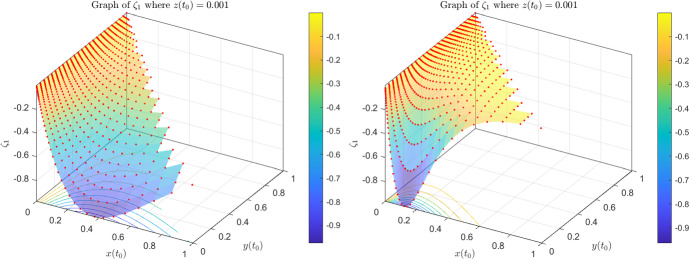
Graph of the function $\zeta _1$ generated by surface interpolation of $(x(t_0), y(t_0))$ (red points) with a fixed value $z(t_0) = 0.001$. Left panel: $\mathcal {R}_0 = 2.7$; Right panel: $\mathcal {R}_0 = 8$. Both panels assume $\eta ^* = 3.0303$, $q^* = 1.6667$, and $t_0 = 0$ (color figure online)

**Fig. 11:**
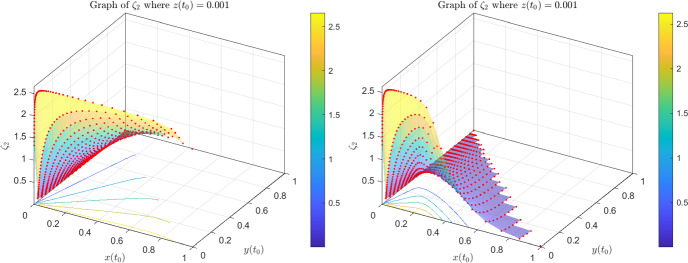
Graph of the function $\zeta _2$ generated by surface interpolation of $(x(t_0), y(t_0))$ (red points) with a fixed value $z(t_0) = 0.001$. Left panel: $\mathcal {R}_0 = 2.7$; Right panel: $\mathcal {R}_0 = 8$. Both panels assume $\eta ^* = 3.0303$, $q^* = 1.6667$, and $t_0 = 0$ (color figure online)

In the same vein, we may consider the numerator $\zeta _1$ and the denominator $\zeta _2$ separately in Figs. 10 and 11. Observe that $\zeta _2$ is not identically constant, hence information from $\zeta _1$ is not sufficient to establish the maximum value of $x_\infty$. Now, both $\zeta _1$ and $\zeta _2$ approach zero for smaller values of $x(t_0)$. Equation (29) implies that $\zeta _1 \rightarrow 0$ as $x(t_0) \rightarrow 0$ independent of $y(t_0)$, $z(t_0)$, and the parameter values. We might observe the same behavior (at least numerically) for $\zeta _2$. However, it remains unknown whether $\zeta _1/\zeta _2$ will converge to some value, assuming $y(t_0)$ and $z(t_0)$ fixed. Further analysis is needed in light of Theorem 4.7.

**Fig. 12:**
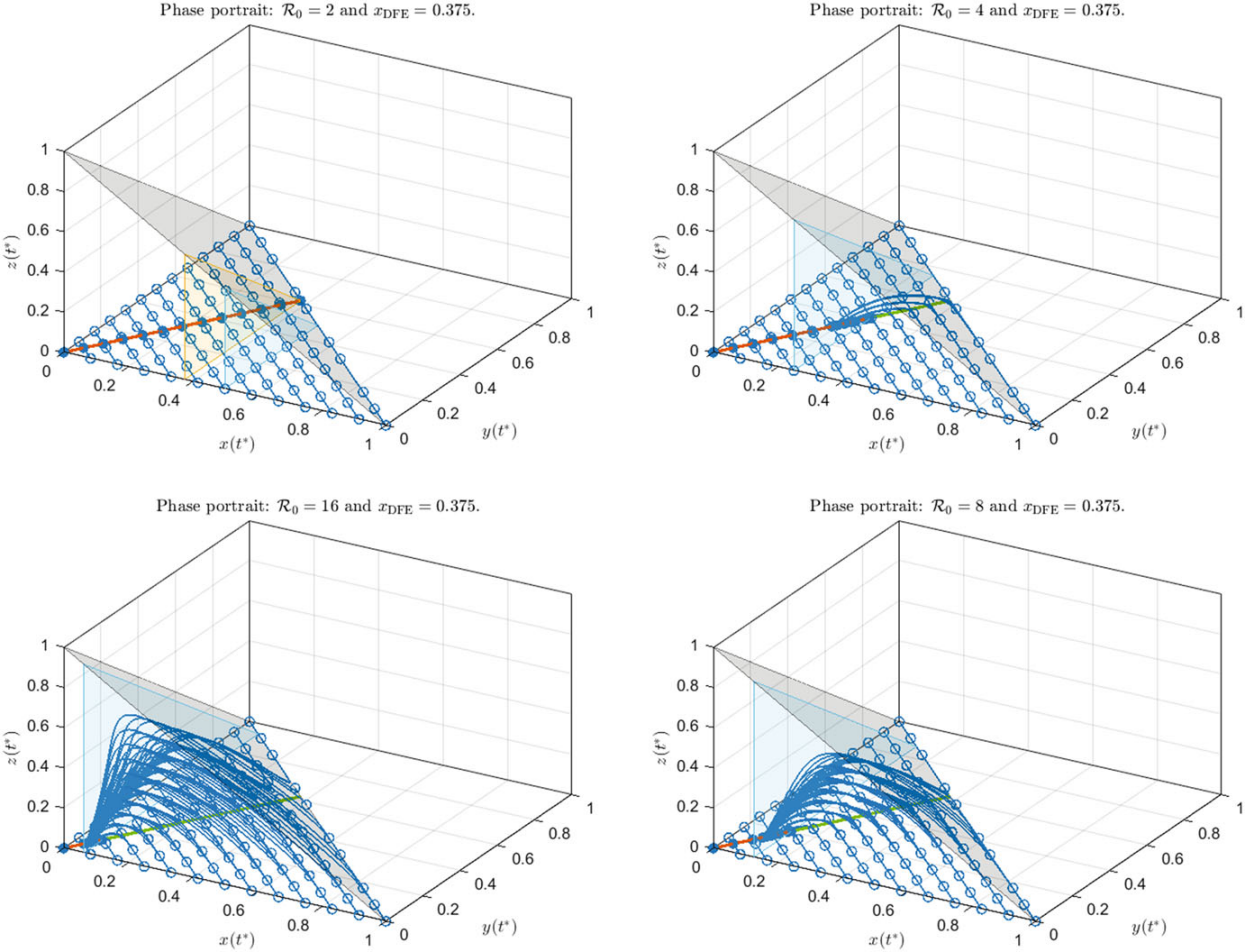
Phase portrait with solutions in the state space satisfying $z(t_0)=0.0001$. Clockwise from top-left: $\mathcal {R}_0 = 2$, $\mathcal {R}_0 =4$, $\mathcal {R}_0 =8$, and $\mathcal {R}_0 = 16$. For all plots, $q^* =5/3$ and $\eta ^* =1/0.33$

Figure 12 depicts solution trajectories when $z(t_0)$ is small. Observe that the final state of each solution, obtained by taking $t^* \rightarrow \infty$, and indicated by a filled blue circle in the phase portrait, is located in the stable set; the previous figures also provide the same results. Hence, after considering additional solutions, we may conclude that the final states will cover the stable set. That is, we may conjecture that **the stable set is the smallest attracting set**. The previous figures also support this claim, where solutions exist that tend to a point close to either the origin or the equilibrium point where $x = \widehat{x}$.

**Fig. 13:**
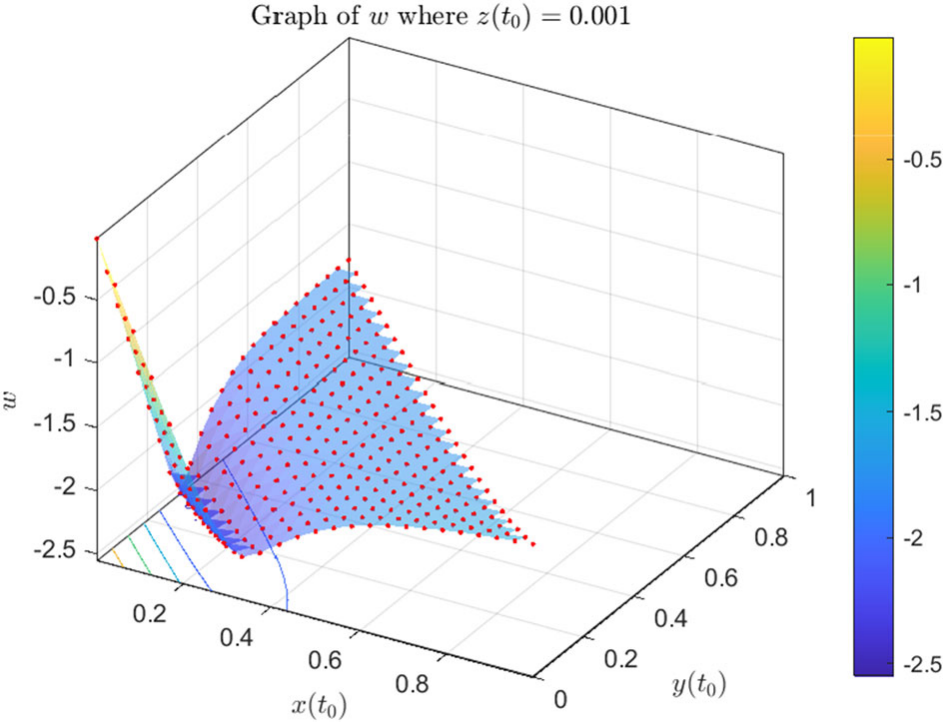
The value of $w = -\mathcal {R}_0(1+q^*)x_\infty$ at different values of $(x(t_0),y(t_0))$ (red points) where $z(t_0) = 0.001$. We generate this figure with $\mathcal {R}_0 = 8$, $\eta ^* = 1/0.33$, $q^* = 5/3$, and $t_0 = 0$ (color figure online)

Figure 13 indicates a possibility that $x_\infty$ emerges from more than one branch of the Lambert function (Remark 4.13). Here, we plot $w = -\mathcal {R}_0(1+q^*)x_\infty$, which satisfies the transcendental equation $we^w = \zeta$. Observe that $w < -1$ over a set of initial points inside the region $0.1< x(t_0) + y(t_0) < 1$ and $z(t_0) = 0.001$, leading to the choice $k = -1$ in the formula $x_\infty = -\mathcal {W}_k(\zeta )/[\mathcal {R}_0(1+q^*)]$. Outside this set, $x_\infty$ takes the principal branch $k = 0$.

From the standpoint of maximizing $x_\infty$, we conjecture from the same figure that the maximum of $x_\infty$ (or equivalently, the minimum of *w*) might not be at a single initial point but along a curve.

### Epidemic peaks

In the context of our model (6), we identify the epidemic peak with the peak in the asymptomatic population (*z*). Figure 12 also sheds light on how the peaks in *z* change with $\mathcal {R}_0$. Increasing $\mathcal {R}_0$ moves the plane $x=1/\mathcal {R}_0$ towards the *yz*-plane, i.e, where $x=0$. This effect permits solutions with $x(t_0)<1/\mathcal {R}_0$ a larger time interval where $z(t^*)$ increases to a local maximum (peak). Furthermore, the peak in *z* increases with $\mathcal {R}_0$.

**Fig. 14:**
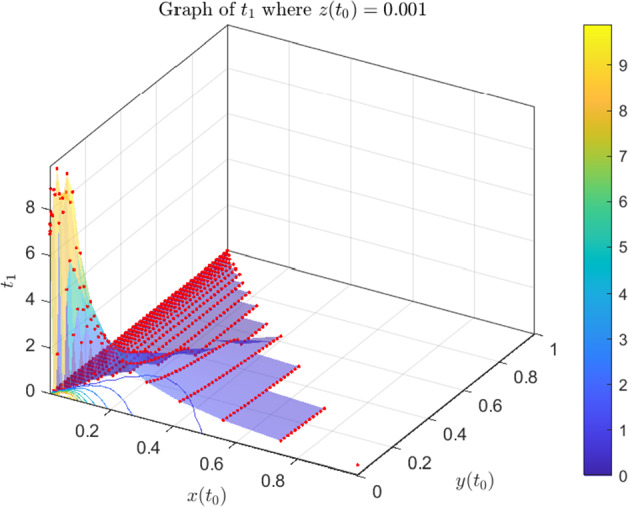
The time that the confined population peaks ($t_1$) as a function of $x(t_0)$ and $y(t_0)$, for $t_0 = 0$ and $z(t_0) = 0.001$. The surface is interpolated from sample points (red) computed from the solutions. Parameter values: $\mathcal {R}_0 = 8$, $\eta ^* = 1/0.33$, and $q^* = 5/3$. The solutions are computed using a stiff solver (ode23s in Matlab) for $0 \le t \le 365$ (color figure online)

Figure 14 below qualitatively indicates that the time $t_1$ the confined population size peaks is a function of the initial condition, where $y/x < q^*$ and $y' > 0$ at $t_0$. Here, we compute $t_1$ as the smallest value such that *y*(*t*) attains its maximum among all numerically computed values of *t*. Our computations also suggest a substantial computed value in $t_1$—implying possible longer times for confined individuals to peak in numbers—for points $(x(t_0), y(t_0))$ near the origin.

In the region $y/x > q^*$, we have $t_1 = t_0$, which agrees with Theorem 4.7(b) where $y'(t) < 0$ for all $t \ge t_0$.

We advise readers to interpret Fig. 14 from a qualitative standpoint due to numerical challenges. Preliminary simulations using a standard non-stiff solver (ode45 in Matlab) revealed minute oscillations for a solution where *y*(*t*) is visually increasing. These oscillations potentially lead to a computed value of $t_1$ that is significantly less than the correct value, thus implying possible erratic behavior near the origin. For this reason, we chose a stiff solver (ode23s) to capture the essential variations in $t_1$ and minimize erratic behavior at the cost of numerical accuracy.

### Solution curves and final-size bounds

**Fig. 15:**
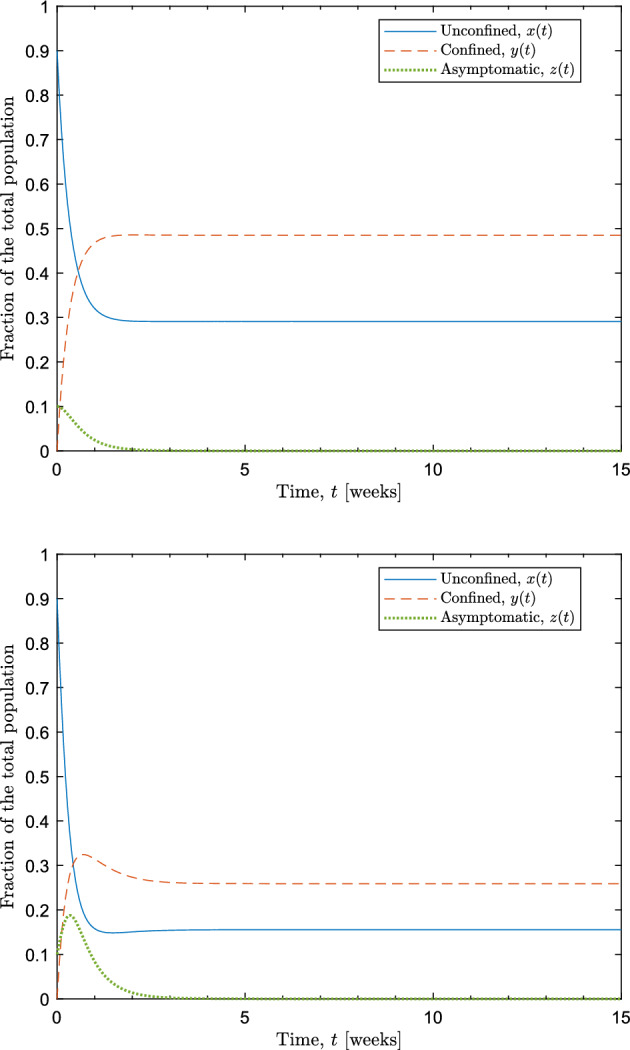
The components of the solution (*x*(*t*), *y*(*t*), *z*(*t*)) of (6) with initial point $(x(0),y(0),z(0)) = (0.9,0,0.1)$, given $\mathcal {R}_0 = 1.1$ (top panel) and $\mathcal {R}_0 = 2.7$. The fraction of the population that is unconfined (blue, solid line) initially decreases and may increase for a sufficiently large $\mathcal {R}_0$ before converging to $x_\infty$. Meanwhile, both the confined (red, dashed line) and asymptomatic population sizes (green, dotted thick line) either follow monotone change or encounter peaks at different times before converging to $y_\infty = q^*x_\infty$ and $z_\infty = 0$, respectively. Both panels are generated with $\eta ^* = 1/0.33$, $q^* = 5/3$ (color figure online)

Figure 15 depicts the behavior of the model (6) with a fixed initial point. Although both panels in this figure consider $\mathcal {R}_0 > 1$, they are differ by the value of $\mathcal {R}_C = \mathcal {R}_0/(1 + q^*)$. Fixing $q^* = 5/3$, we observe the following:

- For $\mathcal {R}_C < 1$ (given by $\mathcal {R}_0 = 1.1$), the confined and asymptomatic populations monotonically increase and decrease, respectively. The unconfined population monotonically decreases.
- If $\mathcal {R}_C > 1$ (given by $\mathcal {R}_0 = 2.7$), both the confined and asymptomatic populations experience peaks before converging to their final sizes. The unconfined population may experience a transient decrease followed by an increase due to the confinement/deconfinement mechanism.

In both cases, the confined fraction is either monotone or experiences a peak, agreeing with Theorem 4.7.

**Fig. 16:**
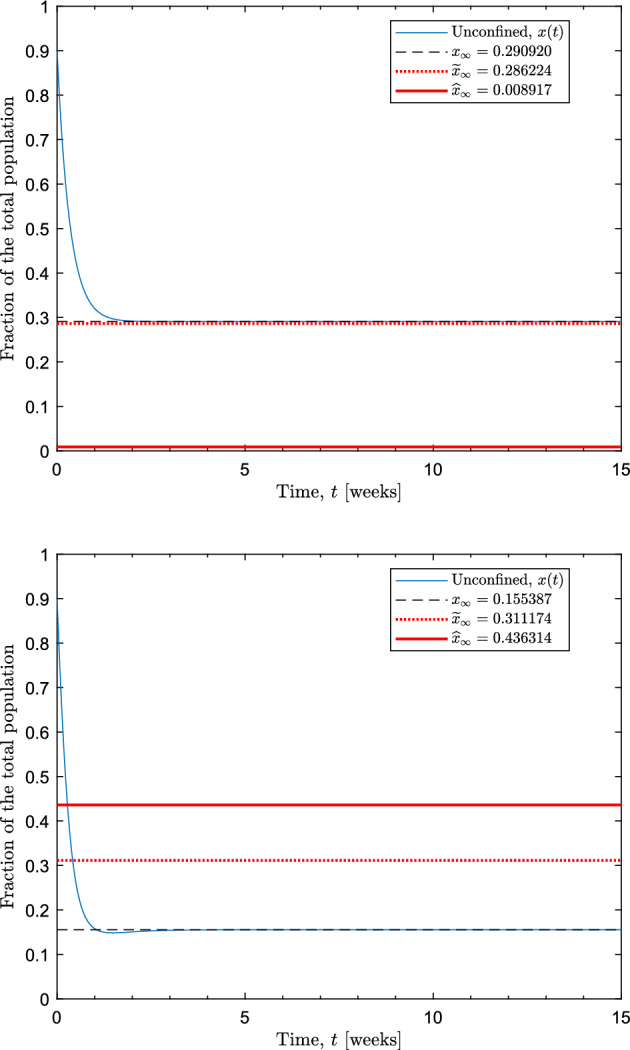
The solution trajectory of unconfined susceptible (*x*(*t*), solid blue line) from the model (6) for $\mathcal {R}_0 = 1.1$ (top panel) and $\mathcal {R}_0 = 2.7$ (bottom panel). This fraction converges towards the final size $x_{\infty }$ (black, dashed horizontal line), which is bounded by $\widetilde{x}_\infty$ (red, thick dotted horizontal line) and $\widehat{x}_\infty$ (red, thick solid horizontal line) according to Corollary B.4. Both panels are generated with $\eta ^* = 1/0.33$, $q^* = 5/3$, and $(x,y,z) = (0.9,0,0.1)$ at initial time $t = 0$ (color figure online)

Figure 16 focuses on the component *x*(*t*), which converges to its final size $x_\infty$. This figure also depicts $\widetilde{x}_\infty$ and $\widehat{x}_\infty$, which may be upper of lower bounds depending on $\mathcal {R}_0$. Observe that $\widetilde{x}_\infty$ provides a better approximation of $x_\infty$ than $\widehat{x}_\infty$.

## Discussion

SARS-CoV-2 crippled our society around the globe, forcing self-isolation and quarantine for many months. In 2020 we proposed a mathematical model study to assist the pandemic in Mexico, the 10th most populous nation, with 1.5 critical care beds per 1,000 people, making it vulnerable to COVID-19. Our report examined Mexico’s COVID-19 pandemic and projects scenarios to assess sharp or gradual quarantine lifting techniques. On June 1, 2020, Mexico abolished rigorous social distancing laws, resulting in pandemic statistics with considerable variations and uncertainty. We performed parameter fitting to the mathematical model, but long-term behavior was not performed.

Here, retaking the modeling work (Ricardo-Azanza and Hernandez-Vargas 2020) we formalize the concept of confinement tonicity to formulate confinement restrictions established during pandemics. Much of our analysis focused on the stability and limiting behavior of model (6). Theorem 4.9 asserts that, irrespective of the parameter values, the model will stabilize to some steady state given by $x = \xi$, $y = q^*\xi$, and $z = 0$, where $0 \le \xi \le \widehat{x}$. This point lies in the stable set $\mathcal {X}^*_\text {st}$ which satisfies local stability according to Theorem 4.12. According to this observation, our model predicts that the infectious population *z* will eventually clear as expected of an acute disease. Moreover, the confined population will eventually reach a level proportional to the number of unconfined, i.e., $y = q^*x$.

An important result is the final size relation

$$\begin{aligned} x_{\infty }e^{-\mathcal {R}_0\,(1+q^*)x_{\infty }}&= \frac{x(t_0)e^{-\mathcal {R}_0[x(t_0)+y(t_0)+z(t_0)]}}{e^{\Omega (p_0, t_0)}} \end{aligned}$$

in (22). Theorem 4.12 leverages this equation to establish an attractive subset $\mathcal {X}^*_{\textit{as}}$ of $\mathcal {X}^*_\text {st}$. Here,

$$\begin{aligned} \Omega (p_0, t_0)&= \int _{t_0}^{\infty } Q(\theta )\,d\theta ,&Q(t)&= q^* - \frac{y(t)}{x(t)} = \frac{y'(t)}{x(t)}, \end{aligned}$$

where the ordered pair $(p_0, t_0)$ corresponds to the solution $\varphi (t) = (x(t), y(t), z(t))$ of (6) with initial condition $\varphi (t_0) = p_0$. We computed the integral $\Omega$ from the expression $q^*x - y = Qx$ associated with the transition between the confined (*y*) and the unconfined (*x*) susceptible compartments. This expression also implies that *x* may not always decrease.

The function *Q*(*t*) can serve as a standalone measurement for the net direction and magnitude of flows between the confined and deconfined susceptible compartments. This prompted us to think of a succint and appropriate term as a reference for future work. Since *Q* shares equal signs with $y'$, having $Q > 0$ indicates net inward flow into the *y* compartment (confinement) and $Q < 0$ indicates the opposite outward flow (deconfinement). Hence in the context of Theorem 4.7, the system may either stick to a single (de)confinement state (where the sign of $y'$ is fixed) or switch only once from confinement to deconfinement before reaching the equilibrium state where $Q = 0$ (Theorem 4.9). We may liken this behavior to osmosis between two chemical solutions, characterized by the diffusion of water from one solution to the other until equilibrium is reached. The *tonicity* of one solution relative to the other determines the direction of osmosis, hence inspiring our term “confinement tonicity” for *Q*. Furthermore, we may call the confined compartment **hypertonic** when $Q > 0$, **hypotonic** when $Q < 0$, and **isotonic** when $Q = 0$.

Further mathematical analysis is required on the integral $\Omega$ in light of the use of the Lambert function. It is desirable to find a *non-integral closed-form expression* of $\Omega$ that leads to an expression of the final size relation (22) that depends *only* on initial values and parameters. This simplification is a potential key step to upgrade Theorem 4.12 by verifying the hypotheses of statements (b) and (c).

However, achieving a tractable form for $\Omega$ or one of its partial derivatives as suggested above may be challenging, unless, for instance, there is an exact solution (Harko et al. 2014) to the model (6). Moreover, we might require auxiliary differential equations involving higher-order derivatives based on the model. Alternatively, we may apply advanced mathematical methods to study partial derivatives of $\Omega$ in the coordinates of $p_0$, allowing us to investigate how $\Omega$ changes with initial conditions. As a first step, we generated graphs of $\zeta _2$ in Fig. 11, noting that $\Omega = \ln (\zeta _2)$.

Nevertheless, we achieved the following bounds for $\zeta$ in Theorem B.3:

$$\begin{aligned} \widetilde{\zeta }&:= \frac{\zeta _1}{e^{\Omega _{\max }(p_0, t_0)}},&\widehat{\zeta }&:= \frac{\zeta _1}{e^{Q(t_0)(t_1-t_0)}}, \end{aligned}$$

where $\zeta < \widetilde{\zeta } \le \widehat{\zeta }$. We also determined the following bounds in Corollary B.4:

$$\begin{aligned} \widetilde{x}_\infty&:= -\frac{\mathcal {W}_k(\widetilde{\zeta })}{\mathcal {R}_0(1+q^*)},&\widehat{x}_\infty&:= -\frac{\mathcal {W}_k(\widehat{\zeta })}{\mathcal {R}_0(1+q^*)}, \end{aligned}$$

where $k = 0$ or $k = -1$, depending on the value of $\mathcal {R}_0(1 + q^*)x_\infty$. The choice of *k* determines the value of $x_\infty$ and its order relationship with the two bounds. As indicated in Fig. 13, the choice of *k* with some fixed parameters will generally depend on the initial points.

The formulas of $\widetilde{x}_\infty$ and $\widehat{x}_\infty$ in (28) provide two different approaches to approximate $x_\infty$, with $\widetilde{x}_\infty$ relying on the definite integral $\Omega (p_0, t_0)$ evaluated between initial and *y*-peak times. On the other hand, $\widehat{x}_\infty$ relies on the solution values at $t_0$ (initial time) and the value of $t_1$ (time where confined population peaks). However, both of these approaches come with two key limitations: (1) these do not apply to the case where $y'(t) > 0$ for all $t > t_0$; (2) the values of $\widetilde{x}_\infty$ and $\widehat{x}_\infty$ are only useful when they are both positive and less than unity.

Unlike $\widetilde{x}_\infty$, which relies on an integral for an accurate bound, $\widehat{x}_\infty$ is practical in the sense that we can use the expression to identify possible disease control measures at the cost of accuracy. Increasing $\widehat{x}_\infty$ as a lower bound of $x_\infty$ favors disease control by minimizing the loss of unconfined individuals to the infectious stage (*z*). By contrast, decreasing $\widehat{x}_\infty$ as an upper bound may indicate a more pronounced effect by the epidemic through reduced final sizes. We note that the initial solution values affect both the numerator and the denominator of $\widehat{x}_\infty$. Thus, subtle changes in the initial condition may increase or decrease $\widehat{x}_\infty$, or even change the role of $\widehat{x}_\infty$ as an upper or a lower bound. These changes carry over to the peak time $t_1$ for the confined population, where a significant increase in $t_1$ may reduce $\widehat{\zeta }$.

We now discuss some attempts to achieve a tractable form of the integral $\Omega (p_0, t_0)$. If *Q* has an antiderivative *F* such that $F'(t) = Q(t)$ for all $t \ge 0$, and if $F_\infty = \lim _{t\rightarrow \infty } F(t)$ exists, then the Fundamental Theorem of Calculus yields

$$\begin{aligned} \Omega&= \lim _{t\rightarrow \infty } \int _{t_0}^t F'(\theta )\,d\theta = \lim _{t\rightarrow \infty }[F(t) - F(t_0)] = F_\infty - F(t_0). \end{aligned}$$

An open problem is to find such *F* that is expressed in the solution coordinates. In a different direction, we may appeal to Taylor expansion and compute the derivatives of *Q*. Denote $f_n = y^{(n)}/x$ and $g = \ln x$ (note that $Q = f_1$ and $g' = x'/x$). Then

$$\begin{aligned} f_n'&= \left( \frac{y^{(n)}}{x}\right) ' = \frac{xy^{(n+1)} - x'y^{(n)}}{x^2} = f_{n+1} - g'f_n,&n&= 1, 2, \dots \end{aligned}$$

That is, the derivative of $f_n$ involves $f_n$ itself, $f_{n+1}$, and $g'$. With $Q = f_1$, we can express $Q^{(n)}$ in terms of $f_1$, $f_2$,..., $f_n$, $f_{n+1}$, and the derivatives $g'$, $g''$,..., $g^{(n)}$. We list the first three derivatives below:

$$\begin{aligned} Q'&= f_2 - g'Q \\ Q''&= f_3 - 2g'f_2 + [(g')^2 - g'']Q \\ Q'''&= f_4 - 3g'f_3 + 3[(g')^2 - g'']f_2 + [3(g')(g'') - g''' - (g')^3]Q \end{aligned}$$

Simplifying these expressions, which would lead to a tractable Taylor expansion, may be achieved from equations involving members of the sequence $\{f_n \mid n = 1, 2,\ldots \}$.

Statements (b) and (c) of Theorem 4.7 imply the existence of $t_1 \ge t_0$ such that the derivative $y'$ assumes a fixed sign over each of the open intervals $(t_0, t_1)$ and $(t_1, \infty )$; in case $t_1 > t_0$, *y* attains a local maximum (peak) at $t_1$. Thus, with $Q = y'/x$, we have

$$\begin{aligned} \int _{t_0}^{\infty } Q(\theta )\,d\theta&= \int _{t_0}^{t_1} \frac{y'(\theta )}{x(\theta )}\,d\theta + \int _{t_1}^{\infty } \frac{y'(\theta )}{x(\theta )}\,d\theta , \end{aligned}$$

where each integral on the right-hand side shares the same sign with $y'$.

Part of our dynamical analysis of (6) involved

$$\begin{aligned} \mathcal {R}_C&= \frac{\mathcal {R}_0}{1 + q^*}, \end{aligned}$$

as defined in Eq. (16) based on the next-generation matrix method. Hence, when the disease has already spread, the model supports the necessity of confinement to control the disease with the goal of $\mathcal {R}_C < 1$ (equivalently $\mathcal {R}_0 < 1 + q^*$). A more aggressive confinement measure is associated with increasing $q^*$, which reduces the value of $\mathcal {R}_C$ while increasing the threshold $\mathcal {R}_0 = 1 + q^*$ where confinement fails to contain the disease.

In contrast, reducing $q^*$ to zero corresponds to a lifting of confinement, from which $\mathcal {R}_C$ increases to $\mathcal {R}_0$. Moreover, the model predicts classic epidemic behavior in the limit where $q^* = 0$ (assuming that $y = 0$). Thus, a more mechanistic model should consider $q^*$ as a time-dependent function. For example, we may choose two constants, $a > 1$ and $T > 0$, and define

$$\begin{aligned} q^*(t^*)&= {\left\{ \begin{array}{ll} a, &{} \text {if }0 \le t^* \le T, \\ 0, &{} \text {if }t^* > T. \end{array}\right. } \end{aligned}$$

Sereno et al. (2021) explored the general form of suspending interventions in SIR-type systems and found that the unconfined (susceptible), $x(q^*)(1+q^*)$, should be smaller than $1/\mathcal {R}_0$ before $t^* = T$, in order to avoid a second wave of infection. Azanza Ricardo and Hernandez-Vargas (2020) explored the effects of different scenarios to lift the confinement, which would correspond to different definitions of $q^*$.

Our model also supports the need for basic measures to curtail transmission (with the goal of reducing $\mathcal {R}_0 < 1$), since it predicts that $\mathcal {R}_C< \mathcal {R}_0 < 1$ at any tolerance for confinement ($q^*$). We recall that $\mathcal {R}_C$ is the basic reproductive number associated with (6) computed by next generation matrix.

We conclude with the following comments. Like many other models, the model (3) was made to explore quarantine as a viable disease control strategy in response to the first stages of the epidemic. Therefore, if the COVID-19 pandemic continues to persist in the long-term, then factors like demography may become necessary. Thus, for example, we may extend the model (3) to

$$\begin{aligned} S'&= (N_S - \mu S) - \frac{\beta E}{N}S - (qS - \tau C), \\ C'&= qS - \tau C - \mu C, \\ E'&= \frac{\beta E}{N}S - \eta E - \mu E, \\ I'&= \varepsilon \eta E - \delta I - \mu I, \\ R'&= (1-\varepsilon )\eta E + \delta I - \mu R, \end{aligned}$$

where $N_S$ is the constant birth/immigration rate, and $\mu$ is the per capita mortality rate. After some tedious computations, we find that this new model admits only two equilibrium points: the disease-free equilibrium where $E = I = R = 0$ and the endemic equilibrium (EE). Furthermore, the basic reproduction number is now given by

$$\begin{aligned} \mathcal {R}_0&= \frac{\beta (\mu + \tau )}{(\eta + \mu )(\mu + \tau + q)}, \end{aligned}$$

and EE exists with all positive coordinates if and only if $\mathcal {R}_0 > 1$. Besides extending the model to account for long-term population dynamics, coupling an in-host model of SARS-CoV-2 (the pathogen behind COVID-19) (Hernandez-Vargas and Velasco-Hernandez 2020) may reveal a deeper understanding on how viral infection together with quarantine measures can help control the disease.

Further analysis may be directed towards characterizing transient dynamics, particularly epidemic peaks, with respect to model parameters. Our mathematical results are guided by this central problem.

## Stability theory

The systems considered here take the form

A1 $$\begin{aligned} \frac{dx}{dt}&= f(x),&x(0)&= x_0, \end{aligned}$$

where *x* (the state vector) is constrained to a state space $\mathcal {X}$ in $\mathbb {R}^n$. A solution $\phi (t, x_0)$ uniquely exists with each initial state (initial point) $x_0 \in \mathcal {X}$ provided the vector field *f* is Lipschitz continuous. In the following results, we consider the system (A1) constrained to its state space $\mathcal {X}$. We also equip $\mathbb {R}^n$ with the Euclidean norm $\Vert \cdot \Vert$ and define the distance between a point *x* and a set $\mathcal {Y}$ as $\Vert x\Vert _\mathcal {Y}:= \inf _{y \in \mathcal {Y}} \Vert x - y\Vert$.

### Definition A.1

(*Equilibrium set*) We can the set $\mathcal {X}^* \subseteq \mathcal {X}$ an **equilibrium set** for the system (A1) if each $x^* \in \mathcal {X}^*$ satisfies $f(x^*) = 0$, from which $\phi (t, x^*) = x^*$ for all $t \ge 0$. Thus, we call $x^*$ an **equilibrium point**.

### Definition A.2

(*Attractivity of an equilibrium set*) We call a closed equilibrium set $\mathcal {X}^* \subseteq \mathcal {X}$ **attractive** in $\mathcal {X}$ if $\lim _{t\rightarrow \infty } \Vert \phi (t, x_0)\Vert _{\mathcal {X}^*} = 0$ for all $x_0 \in \mathcal {X}$.

The existence of an attractive subset of $\mathcal {X}^*$ suffices for $\mathcal {X}^*$ to be attractive, hence it is crucial to determine the **smallest attractive subset** $\mathcal {Y} \subseteq \mathcal {X}$ such that the only attractive set $\mathcal {Z} \subseteq \mathcal {Y}$ is $\mathcal {Z} = \mathcal {Y}$.

### Definition A.3

(*Local stability of an equilibrium set*) We call a closed equilibrium set $\mathcal {X}^* \subseteq \mathcal {X}$ **locally stable** in $\mathcal {X}$ if, for all $\epsilon > 0$ there exists $\delta > 0$ such that every point $x_0$ with $\Vert x\Vert _{\mathcal {X}^*} < \delta$ satisfies $\Vert \phi (t, x_0)\Vert _{\mathcal {X}^*} < \epsilon$ for all $t \ge 0$.

### Definition A.4

(*Asymptotic stability of an equilibrium set*) A closed equilibrium set $\mathcal {X}^* \subseteq \mathcal {X}$ is **asymptotically stable** in $\mathcal {X}$ if $\mathcal {X}^*$ is both locally stable and attractive in $\mathcal {X}$.

### Theorem A.5

(Lyapunov theorem Khalil and Grizzle 2002; Haddad and Chellaboina 2011) Consider the system (A1) constrained to the state space $\mathcal {X}$ with an equilibrium point $x^*$ contained in $\mathcal {Y} \subseteq \mathcal {X}$. Suppose that there exists a **Lyapunov funcion** $V: \mathcal {Y} \rightarrow \mathbb {R}$ defined by the following properties: $V(x) > 0$ for all $x \ne x^*$, $V(x^*) = 0$, and $dV/dt = \nabla V(x) \cdot f(x) \le 0$. Then $x^*$ is locally stable in $\mathcal {X}$. If we also have $\dot{V}(x) < 0$ for all $x \ne x^*$, and $\dot{V}(x^*) = 0$, then $x^*$ is asymptotically stable.

## Final size bounds

The value of $\zeta$ in Eq. (23) is specific to the solution of (6) at a given initial time $t_0$ and the corresponding initial point $p_0 = (x(t_0), y(t_0), z(t_0))$. Indeed, this value will depend on the integral

$$\begin{aligned} \Omega (p_0, t_0)&:= \int _{t_0}^{\infty } Q(\theta )\,d\theta ,&Q(t)&= q^* - \frac{y(t)}{x(t)} = \frac{y'(t)}{x(t)}. \end{aligned}$$

By the continuous dependence of (6) with initial conditions, the ordered pair $(p_0, t_0)$ determines the specific trajectories of both *x*(*t*) and *y*(*t*), which in turn determines the *exact* value of $\Omega$.

Finding a closed-form expression for $\Omega (p_0, t_0)$ is still an open problem (see the Discussion, Sect. 6 for an explanation). Nevertheless, it is possible to obtain closed-form bounds for $\Omega$ even if we only know that $x_\infty$ is (up to a constant multiple) the value of some branch of the Lambert function.

To this end, we fix $(p_0, t_0)$ and impose the following hypotheses:

1. $$z(t_0) > 0$$ and $$x(t_0) > 0$$.
2. $y'(t_0) < 0$ or there exists $t_1 \ge t_0$ such that $y'(t_1) = 0$.
3. The improper integral $\Omega (p_0, t_0)$ exists.

Then by the positive invariance of $\mathcal {Y}_3$ (Theorem 4.2), hypothesis (H1) implies that $x(t) > 0$ and $z(t) > 0$ for all $t \ge t_0$, ensuring that *Q* is defined on the interval $[t_0, \infty )$. With hypothesis (H2), Theorem 4.7 ensures the existence of a unique $t_1 \ge t_0$ defined as follows:

- If $y'(t_0) < 0$, then *y* strictly decreases over $[t_0, \infty )$; here, we let $t_1 = t_0$.
- If $y'(t_0) \ge 0$, then let $t_1$ be the unique value such that $y'(t_1) = 0$, hence $y'(t)$ shares the same sign with $t_1 - t$ over $[t_0, \infty )$.

Hence, *y* has a unique global maximum (or “peak”) $y_{\max }:= y(t_1)$ over $[t_0, \infty )$. Please note that $y_{\max }$ is based on a fixed $(p_0, t_0)$. Finally, let us define the area function

$$\begin{aligned} \Omega _t(p_0, t_0)&:= \int _{t_0}^{t} Q(\theta )\,d\theta ,&t&\ge t_0. \end{aligned}$$

Then hypothesis (H3) means that $\Omega (p_0, t_0)$ has a finite limit value, namely

$$\begin{aligned} \Omega (p_0, t_0)&= \lim _{t\rightarrow \infty } \Omega _t(p_0, t_0), \end{aligned}$$

where $(p_0, t_0)$ was fixed.

We remark that our choice of $t_1$ is also the smallest value in $[t_0, \infty )$ such that $y'(t_1) \le 0$, from which $y' > 0$ over $[t_0, t_1]$. Since *Q* shares the same sign with $y'$, we can compute $\Omega (p_0, t_0)$ as

$$\begin{aligned} \Omega (p_0, t_0)&= \int _{t_0}^{t_1} Q(\theta )\,d\theta + \int _{t_1}^{\infty } Q(\theta )\,d\theta \end{aligned}$$

where the integral terms over $[t_0, t_1]$ and $[t_1, \infty )$ are non-negative and negative, respectively.

### Lemma B.1

Fix $(p_0, t_0)$ and consider the solution of (6) with initial point $p_0 = (x(t_0), y(t_0), z(t_0))$. Assume hypotheses (H1)–(H3) and define the following integrals:

$$\begin{aligned} \Omega _{\max }&:= \int _{t_0}^{t_1} Q(\theta )\,d\theta ,&\Omega _{\min }&:= \int _{t_1}^{\infty } Q(\theta )\,d\theta . \end{aligned}$$

Then the following statements hold:

- If $y'(t_0) \le 0$, then $\Omega _{\max } = 0$ and $\Omega = \Omega _{\min } < 0$.
- If $$y'(t_0) > 0$$, then $$\Omega _{\min }< \Omega < \Omega _{\max }$$.

Therefore, $\Omega _{\min } \le \Omega < \Omega _{\max }$ with strict inequality if and only if $y'(t_0) > 0$.

### Proof

Note that $\Omega = \Omega _{\max } + \Omega _{\min }$ where $\Omega _{\max } \ge 0$ (with equality if and only if $t_1 = t_0$) and $\Omega _{\min } < 0$. Hence, we argue as follows:

- If $y'(t_0) \le 0$, then we have $t_1 = t_0$ and $\Omega _{\max } = 0$. Hence, $\Omega = \Omega _{\min } < 0$.
- If $y'(t_0) > 0$, then we must have $t_1 > t_0$ and $\Omega _{\max } > 0$; thus, $\Omega > \Omega _{\min }$. Since $\Omega _{\min } < 0$, we can add both sides with $\Omega _{\max }$ to yield $\Omega < \Omega _{\max }$. Therefore, $\Omega _{\min }< \Omega < \Omega _{\max }$.

We have proven statements (a) and (b), which cover all possible signs of $y'(t_0)$. Hence, their respective conclusions can be recast as a single result, namely that $\Omega _{\min } \le \Omega < \Omega _{\max }$ with strict inequality if and only if $y'(t_0) > 0$. $\square$

### Lemma B.2

Fix $(p_0, t_0)$ and consider the solution of (6) with initial point $p_0 = (x(t_0), y(t_0), z(t_0))$. Assume hypotheses (H1)-(H3). Then

$$\begin{aligned} Q(t_1)(t_1-t_0) \le \Omega _{\max } \le Q(t_0)(t_1-t_0). \end{aligned}$$

### Proof

Consider the sign of $x'$, $y'$, and $Q'$ restricted to the closed interval $[t_0, t_1]$. Then $y' > 0$ by definition of $t_1$. With (H1) ensuring positive values of *x* and *z*, we also have

$$\begin{aligned} x'&= - \eta ^*\mathcal {R}_0xz - y' < 0, \end{aligned}$$

which yields

$$\begin{aligned} Q' = \frac{yx' - xy'}{x^2} \le -\frac{xy'}{x^2} < 0. \end{aligned}$$

Therefore, $Q(t_1) \le Q(t) \le Q(t_0)$ for all $t \in [t_0, t_1]$. Integrating over $[t_0, t_1]$, we have $Q(t_1)(t_1 - t_0) \le \Omega _{\max } \le Q(t_0)(t_1 - t_0)$. $\square$

We are now ready to establish a bound for $x_\infty$ for a fixed $(p_0, t_0)$. Recall Eq. (23) for $\zeta$ and Eq. (27) for $\widetilde{\zeta }$ and $\widehat{\zeta }$.

### Theorem B.3

Fix $(p_0, t_0)$ and consider the solution of (6) with initial point $p_0 = (x(t_0), y(t_0), z(t_0))$. Assume hypotheses (H1)–(H3). Then $\zeta < \widetilde{\zeta } \le \widehat{\zeta }$.

### Proof

By Lemmas B.1 and B.2, under any sign of $y'(t_0)$, we have:

$$\begin{aligned} \Omega (p_0, t_0)&< \Omega _{\max }(p_0, t_0) \le Q(t_0)(t_1-t_0) \\ e^{\Omega (p_0, t_0)}&< e^{\Omega _{\max }(p_0, t_0)} \le e^{Q(t_0)(t_1-t_0)} \\ \frac{1}{e^{\Omega (p_0, t_0)}}&> \frac{1}{e^{\Omega _{\max }(p_0, t_0)}} \ge \frac{1}{e^{Q(t_0)(t_1-t_0)}}. \end{aligned}$$

Multiplying both sides of the last inequality by $\zeta _1$ we get $\zeta < \widetilde{\zeta } \le \widehat{\zeta }$. $\square$

### Corollary B.4

Fix $(p_0, t_0)$ and consider the solution of (6) with initial point $p_0 = (x(t_0), y(t_0), z(t_0))$. Assume hypotheses (H1)–(H3) together with $\zeta > -e^{-1}$, and suppose that

$$\begin{aligned} x_\infty&= -\frac{\mathcal {W}_k(\zeta )}{\mathcal {R}_0(1+q^*)}, \end{aligned}$$

where $$k \in \{0, -1\}$$. Let

$$\begin{aligned} \widetilde{x}_\infty&= -\frac{\mathcal {W}_k(\widetilde{\zeta })}{\mathcal {R}_0(1+q^*)},&\widehat{x}_\infty&= -\frac{\mathcal {W}_k(\widehat{\zeta })}{\mathcal {R}_0(1+q^*)}. \end{aligned}$$

See Eq. (28). Then $x_\infty > \widetilde{x}_\infty \ge \widehat{x}_\infty$ for $k = 0$, and $x_\infty < \widetilde{x}_\infty \le \widehat{x}_\infty$ for $k = -1$.

### Proof

Theorem B.3 yields

$$\begin{aligned} -e^{-1}< \zeta&< \widetilde{\zeta } \le \widehat{\zeta } < 0, \end{aligned}$$

which defines $\mathcal {W}_k$ at $\zeta$, $\widetilde{\zeta }$, and $\widehat{\zeta }$. Since the principal branch $\mathcal {W}_0$ increases while the branch $\mathcal {W}_{-1}$ decreases, we have $x_\infty > \widetilde{x}_\infty \ge \widehat{x}_\infty$ for $k = 0$ and $x_\infty < \widetilde{x}_\infty \le \widehat{x}_\infty$ for $k = -1$. $\square$
